# NeuroGeM, a knowledgebase of genetic modifiers in neurodegenerative diseases

**DOI:** 10.1186/1755-8794-6-52

**Published:** 2013-11-14

**Authors:** Dokyun Na, Mushfiqur Rouf, Cahir J O’Kane, David C Rubinsztein, Jörg Gsponer

**Affiliations:** 1 Department of Biochemistry and Molecular Biology, Centre for High-throughput Biology, University of British Columbia, 2125 East Mall, Vancouver, BC V6T 1Z4, Canada; 2 Department of Computer Science, University of British Columbia, 2366 East Mall, Vancouver, BC V6T 1Z4, Canada; 3 Department of Genetics, University of Cambridge, Downing Street, Cambridge CB2 3EH, UK; 4 Department of Medical Genetics, University of Cambridge, Cambridge Institute for Medical Research, Addenbrooke’s Hospital, Hills Road, Cambridge, CB2 0XY, UK; 5 Present address: School of Integrative Engineering, Chung-Ang University, 84 Heukseok-ro, Dongjak-gu, Seoul 156-756, Republic of Korea

**Keywords:** Neurodegenerative diseases, Genetic modifiers, Database, Knowledgebase, Alzheimer’s disease, Parkinson’s disease, Huntington’s disease

## Abstract

**Background:**

Neurodegenerative diseases (NDs) are characterized by the progressive loss of neurons in the human brain. Although the majority of NDs are sporadic, evidence is accumulating that they have a strong genetic component. Therefore, significant efforts have been made in recent years to not only identify disease-causing genes but also genes that modify the severity of NDs, so-called genetic modifiers. To date there exists no compendium that lists and cross-links genetic modifiers of different NDs.

**Description:**

In order to address this need, we present NeuroGeM, the first comprehensive knowledgebase providing integrated information on genetic modifiers of nine different NDs in the model organisms *D. melanogaster*, *C. elegans*, and *S. cerevisiae*. NeuroGeM cross-links curated genetic modifier information from the different NDs and provides details on experimental conditions used for modifier identification, functional annotations, links to homologous proteins and color-coded protein-protein interaction networks to visualize modifier interactions. We demonstrate how this database can be used to generate new understanding through meta-analysis. For instance, we reveal that the *Drosophila* genes DnaJ-1, thread, Atx2, and mub are *generic modifiers* that affect multiple if not all NDs.

**Conclusion:**

As the first compendium of genetic modifiers, NeuroGeM will assist experimental and computational scientists in their search for the pathophysiological mechanisms underlying NDs. http://chibi.ubc.ca/neurogem.

## Background

Intracellular protein aggregation is a feature of many late-onset neurodegenerative diseases (NDs), also called proteinopathies. These include Alzheimer’s disease (AD), Parkinson’s disease (PD), and nine polyglutamine expansion diseases exemplified by Huntington’s disease (HD). The pathophysiology of NDs is very complex, which is one of the reasons why there are no effective strategies that slow or prevent neurodegeneration.

In recent years, significant efforts have been made to identify genes that modify the severity of NDs. Altering the activities of these *genetic modifier* genes on their own may not result in obvious phenotypes in the absence of the conditioning (neurodegeneration-causing) mutation. However, the identified genetic modifiers allow the characterization of biological pathways that modulate the disease and, in some cases, discovery of tractable therapeutic targets. The identification of genetic modifiers has been facilitated due to the development of *in vivo* models of different proteinopathies in organisms such as *D. melanogaster* and *C. elegans*[1-3]. Moreover, genome-wide screens for genetic modifiers have become possible because of high-throughput technologies such as RNA interference [4] or public availability of various transgenic stocks covering most genes such as fly stocks with P-element insertion mutations [5].

Nevertheless, due to the complex nature of the pathological processes underlying proteinopathies, there are large inconsistences in the collected data. Even more importantly, data alone without knowledge or integration into existing databases is bound to remain inaccessible and thus cannot be utilized by the broad scientific community. An integrated database of genetic modifiers of NDs would assist computational and experimental scientists alike in improving their approaches to discover what is common to and distinct for different proteinopathies.

In order to address this need, we assembled the first comprehensive database of genetic modifiers in NDs. NeuroGeM (‘neurodegenerative disease genetic modifiers database’) catalogues and cross-links genetic modifiers of 9 different NDs in three different model organisms, and associates them with information on protein function and other annotations. NeuroGeM contains detailed information on the experimental conditions in which the modifiers were identified, displays the protein-protein interaction sub-network around modifiers, and provides search and display tools to deduce testable hypotheses. Furthermore, in order to demonstrate the broad applicability of the data and tools provided by NeuroGeM, we present the results of a first meta-analysis.

## Construction and content

### Data collection

**Figure 1:**
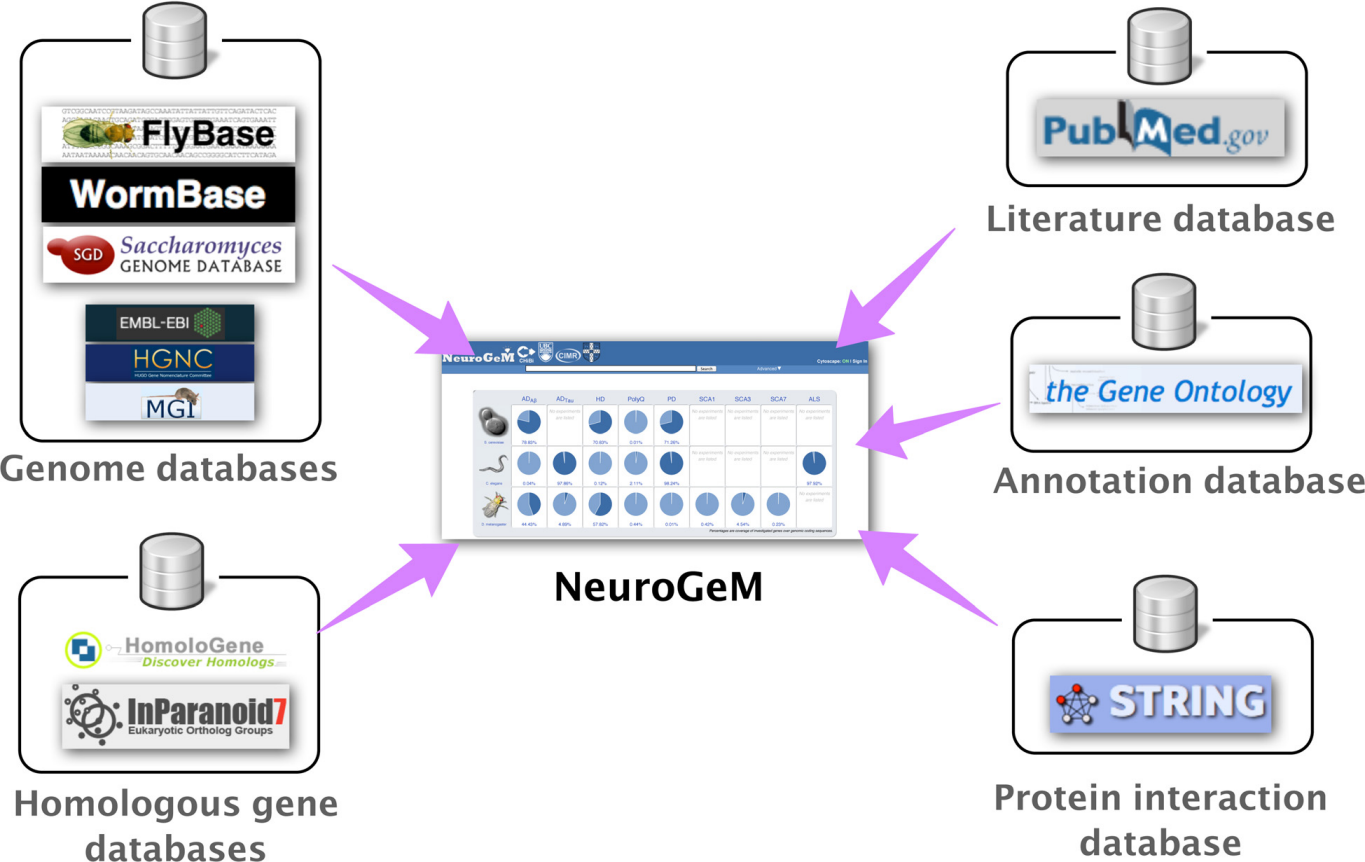
**The contents of NeuroGeM.** NeuroGeM is a comprehensive collection of genetic modifiers of ND models in *D. melanogaster*, *C. elegans*, and *S. cerevisiae*. In order to provide comprehensive information on genetic modifiers, NeuroGeM also integrates information from genome databases (FlyBase, WormBase, SGD, EBML, HGNC, and MGI), the protein interaction database STRING, GeneOntology, and homologous gene databases (HomoloGene and InParanoid). The statistics of the data currently available in NeuroGeM is shown in Table 1, and all terms used in the database are listed in Table 2.

NeuroGeM is a comprehensive collection of literature data on genetic modifiers of NDs and associated genetic information from a variety of databases (Figure 1). The ND models include AD subclassified as AD_Aβ_ (amyloid-beta models) and AD_Tau_ (tau models), HD, PD, Spinocerebellar ataxia type 1, 3 and 7 (SCA1, SCA3, SCA7), Amyotrophic lateral sclerosis (ALS) and generic polyQ-induced disease in *D. melanogaster*, *C. elegans*, and *S. cerevisiae*. At the time of data compilation, all known high-throughput (HT) screens for modifiers carried out in the model organisms and a handful of low-throughput (LT) experimental results were included. Overall, NeuroGeM contains 87,864 experimental records (3,618 for modifiers and 84,246 for non-modifiers) from the 9 different disease models in the three different species (Table 1).

**Table 1:** Statistics of genetic modifiers in NeuroGeM

**Species**	**Disease models**	**Experimental records**		**Modifiers**^**1**^			**Gene coverage**^**2 **^**(%)**
		**Positive records**	**Negative records**	**Enhancers**	**Suppressors**	**Non-modifiers**	
*D. melanogaster*	AD_Tau_	144	571	65	60	549	4.89
	AD_Aβ_	61	6062	25	22	6059	44.43
	PD	1	-	-	1	-	0.01
	HD	1260	8142	130	90	7732	57.82
	SCA1	66	24	16	21	21	0.42
	SCA3	623	52	55	520	49	4.54
	SCA7	14	19	8	5	18	0.23
	PolyQ	22	44	7	10	43	0.44
*C. elegans*	AD_Tau_	75	15909	-	75	15899	97.86
	AD_Aβ_	6	-	1	5	-	0.04
	HD	22	2	1	17	2	0.12
	PD	290	18121	-	268	15767	98.24
	ALS	168	15909	88	80	15817	97.92
	PolyQ	459	40	152	195	20	2.11
*S. cerevisiae*	AD_Aβ_	106	5262	18	23	5248	78.83
	HD	82	9422	28	54	4670	70.83
	PD	216	4699	22	181	4577	71.26
	PolyQ	1	-	-	1	-	0.01

^1^Certain genes have been identified independently as both an enhancer and suppressor under different experimental conditions.

^2^With respect to the protein coding genes.

Importantly, we continually update the knowledgebase with newly published results. In addition, users can submit their own data upon request of a login and the uploaded data will be made accessible to all users after curation (see Additional file 1).

In order to provide comprehensive information on genetic modifiers, we integrated relevant data from other sources into NeuroGeM. All entries in NeuroGeM contain information on gene function/annotations and are accompanied by direct links to the relevant information on FlyBase for *D. melanogaster* (ver Feb 2012) [6], WormBase for *C. elegans* (ver WS230) [7] and SGD for *S. cerevisiae* (downloaded in Jan 2012) [8]. Each gene entered in NeuroGeM contains an ID that is identical to the primary ID of the gene in its respective genome database (FlyBase, WormBase, or SGD). These IDs will allow users to easily access other databases and avoid the effort required for ID conversion. NeuroGeM records have a link to the PubMed entry of the original study from which the records stem.

As protein-protein interaction networks allow identification of functionally associated proteins or important functional clusters [9,10], NeuroGeM visualizes the protein interaction sub-network around a queried gene; each protein node in the network is color-coded according to the available experimental results deposited in NeuroGeM. For this feature, NeuroGeM utilizes the protein interaction data from STRING (ver 9.05) [11]. In order to facilitate the identification of genetic modifiers with the same function or involved in the same process, NeuroGeM provides an ontology-based search functionality that searches for genes by GeneOntology (GO) annotations and the hierarchical structure of GO terms [12].

As users might be interested in finding homologs of the genetic modifiers entered in NeuroGeM, we also integrated homologous gene data from NCBI HomoloGene (build 65) [13] and InParanoid (ver7) [14,15]. Orthologs are defined as genes in different species that have evolved from a common ancestral gene, while paralogs are genes related by duplication within the same species that often have different functions. Homologs are either paralogs or orthologs (for details see NCBI HomoloGene (build 65) [13] and InParanoid (ver7) [14,15]). The homology data covers not only the three model organisms but also *H. sapiens* and *M. musculus*, though no modifiers from these organisms are deposited in NeuroGeM yet. Cross-linking genes *via* homology should facilitate the expansion of modifier studies in other organisms; for instance, confirmation of important modifiers in higher organisms. Gene information, GO annotations and protein interaction data for human and mouse genes were also integrated to help users search for modifiers that are homologous to human and mouse genes of interest. (source: EMBL Rel 68 [16], HGNC downloaded in Jan 2013 [17]; EMBL Rel 68 [18]; and MGI downloaded in Dec 2012 [19]). As detailed information on genes and proteins is frequently updated in their source databases, NeuroGeM also provides links to those databases when available.

### Database implementation

The database was implemented with a web interface compatible with common web browsers to provide access to researchers. The data is stored in a relational database using a MySQL 5.0.59 server. Data processing and HTML generation for displaying information are carried out using PHP 5.3.3. Javascript and AJAX technology are used to improve search functions. CytoscapeWeb [20] is employed to visualize protein-protein interaction networks. The current database is running on Redhat Linux 5.6 with an Apache server 2.2.3. All the data in NeuroGeM can be downloaded as plain text files.

## Utility and discussion

**Figure 2:**
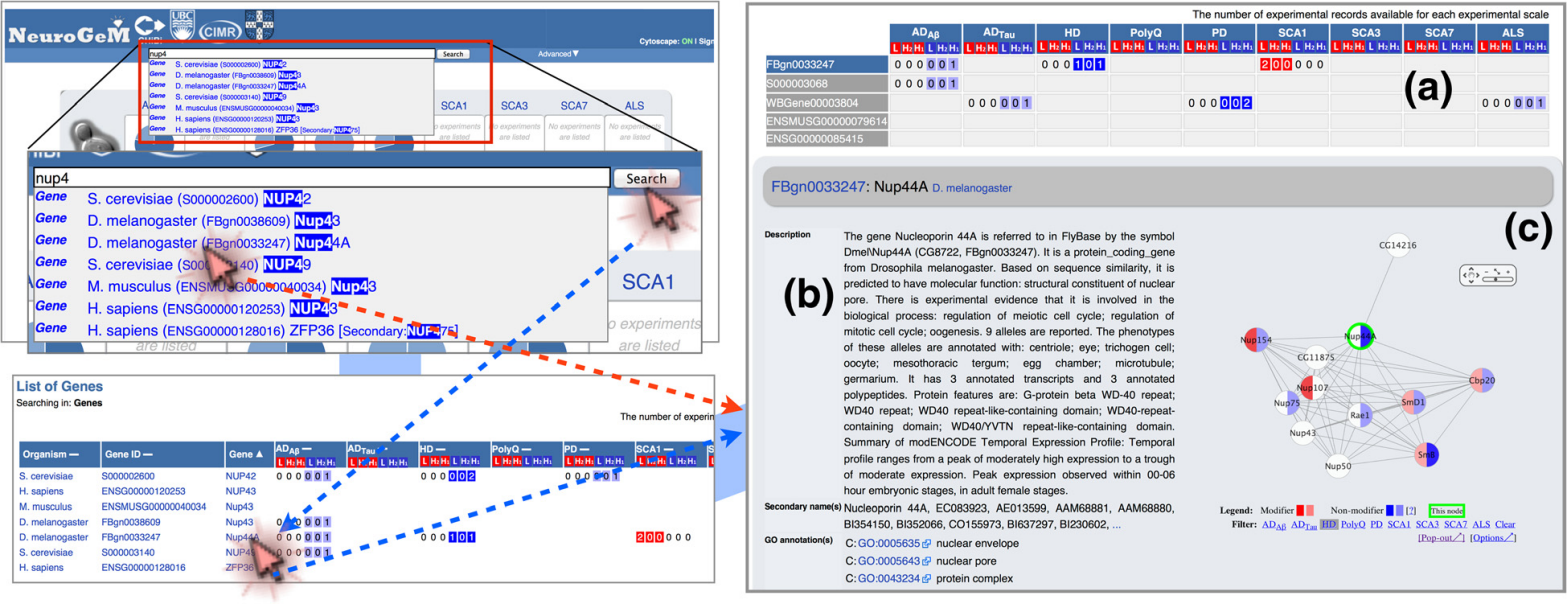
**Search to access information on genetic modifiers in NeuroGeM.** This figure illustrates a keyword-based search to get information of a specific genetic modifier. Other search methods are available in Additional file 2: Figure S1 (categorical search) and Additional file 3: Figure S2 (ontology-based search). The genetic modifier information page includes **(a)** a report summary of experimental records entered in NeuroGeM, **(b)** general information on the gene with links to relevant databases, and **(c)** the protein-protein interaction sub-network around this gene. Details about experimental parameters and results are also shown (Figure 3). The homologous genes section (not shown in this figure) lists genetic information and experimental details of homologous genes.

**Figure 3:**
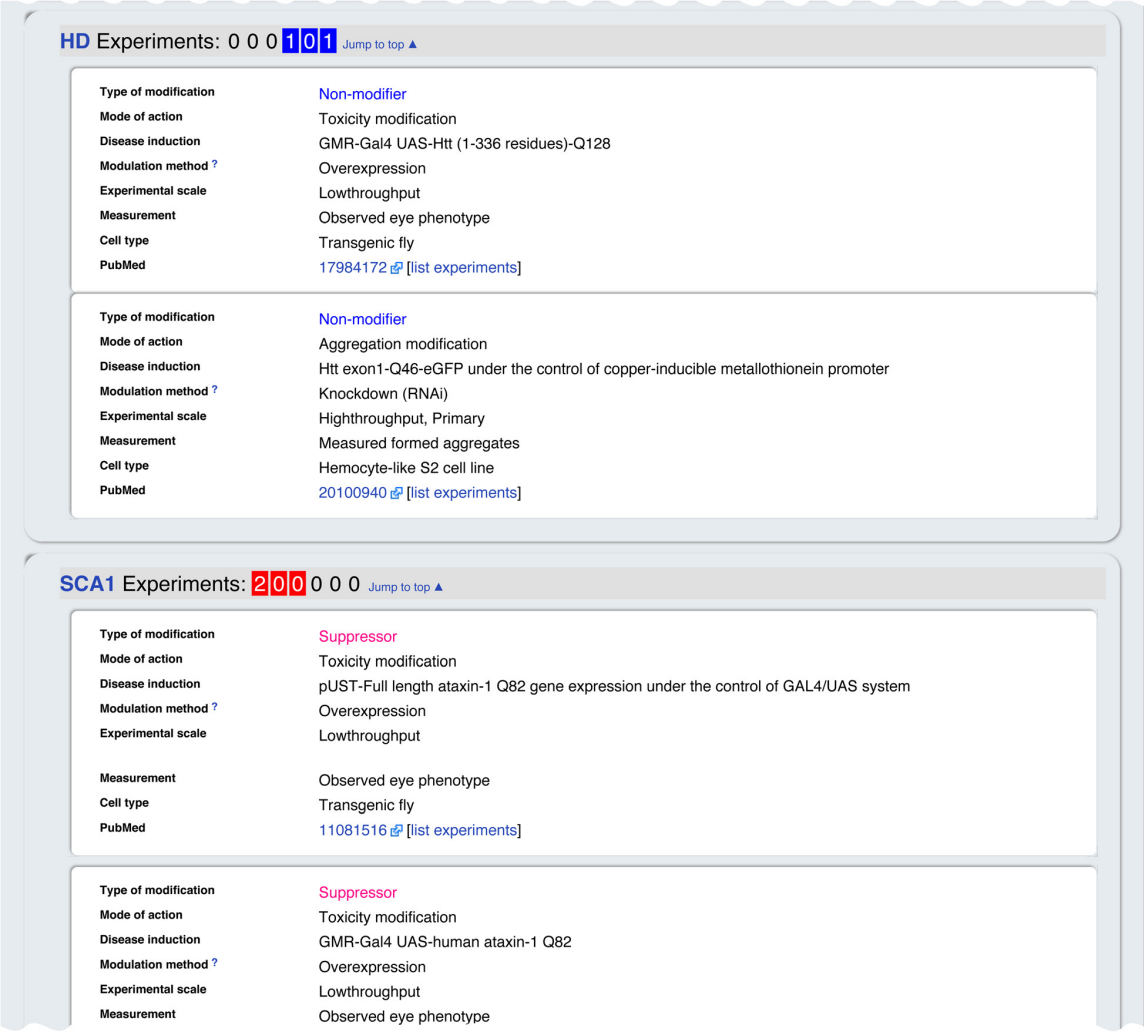
**Details of the experiments that identified the genetic modifiers.** This section of the genetic modifier information page includes the detailed results of the modifier screens (whether the gene is a suppressor, enhancer, or non-modifier), experimental details (mutated genes, method for gene expression modulation, phenotype observation, etc.), and a link to the original article in which the experiment was described (PubMed).

NeuroGeM allows users to access the integrated data in three different ways: (i) a categorical search, (ii) a keyword search, and (iii) an ontology-based search. All search methods take users first to a list of publications or a list of genes that fit the search criteria, from which users can select a gene of interest and consult its modifier information page. Figures 2 and 3 and Additional file 2: Figures S1 and Additional file 3: Figure S2 illustrate the three ways to search genetic modifiers in NeuroGeM and show detailed information on genetic modifiers provided by NeuroGeM.

### Categorical search for studies that identified modifiers

On the front page of the NeuroGeM web site, research results are organized according to disease and model-organism categories: studies that identified modifiers were categorized according to model organism (n = 3) and diseases (n = 9 including two AD subtypes) (Additional file 2: Figure S1). Clicking on an organism icon or disease-specific icon directs the user to a list of publications that belong to that category. Clicking on a pie chart at the intersection of a specific model organism and disease type directs the user to a list of publications that searched for modifiers in the corresponding disease model and organism. Clicking on a specific publication then directs the user to a list of genes that have been tested in that study.

For instance, clicking on the pie chart at the intersection of *D. melanogaster* and HD (Additional file 2: Figure S1) lists 24 publications in which genetic modifiers of HD have been identified in *D. melanogaster*. Clicking on the PubMed ID 17984172 will open a web page that lists the 26 genes that have been examined in this specific study and provides a summary of the experimental records deposited in NeuroGeM for each of these 26 genes (see below for the detailed explanation of the record summary). Clicking on the gene name Nup44A will then direct the user to the genetic modifier information page of Nup44A, which contains the details of the results of different genetic modifier screens for this gene as well as additional information that is discussed in further detail in the “*Genetic modifier information”* section below.

### Keyword-based search for specific genes

NeuroGeM also provides different search methods from a unified search box (Figure 2). As a user starts typing letters in the search box, NeuroGeM suggests matching keywords (gene name, synonyms, IDs, GO terms, *etc.*). If the user selects one of the suggestions, NeuroGeM will open this gene’s modifier information page (Figure 2, red arrow). If the user presses the ‘Search’ button instead, NeuroGeM will list all genes that contain the keyword in their names, synonyms, or IDs. Then, the user can open a specific genetic information page by clicking on one of the listed genes (Figure 2, blue arrow). For example, when typing ‘nup4’ in the search box, several genes that contain the keyword ‘nup4’ are suggested. Selecting the *D. melanogaster* Nup44A gene from the suggestions will open this gene’s page directly. On the other hand, entering ‘nup4’ and clicking the ‘Search’ button will list all genes that contain the keyword and show respective experimental record summaries. The user can open the Nup44A gene page by clicking on the gene name Nup44A of the listed genes.

### Ontology-based search for related genes

The keyword-based search directs the user to a specific gene, but this feature is not appropriate when searching for functionally related genes, *e.g.* searching for genes involved in the *cell cycle*. For such a relation-based search, we provide the ontology-based search. To use the ontology-based search, the user has to type a GO term or GO ID in the search box. Just like the keyword search, suggestions will pop up and the user can select one of the suggested GO terms. Then, NeuroGeM searches for not only genes with the user-specified GO term but also genes with a related (child) term. This ontology-based search will assist users in the identification of modifiers that are associated with specific cellular functions or processes.

For instance, as shown in Additional file 3: Figure S2, the query of ‘*cell cycle* (GO:0007049)’ in *D. melanogaster* returns 656 genes that are involved in the cell cycle: 622 out of 656 genes have been evaluated experimentally, and 256 out of 622 genes have been identified as modifiers. *Drosophila*’s Nup44A gene is also shown in the gene list since Nup44A has GO annotations for *regulation of mitotic cell cycle* (GO:0007346) and *regulation of meiotic cell cycle* (GO:0051445), which are child terms of *cell cycle*. Clicking on the name of Nup44A will direct the user to Nup44A genetic modifier information page.

### Genetic modifier information

At the end of each search, NeuroGeM directs users to the genetic modifier information page of a specific gene. As an example, the genetic modifier information page of the *Drosophila* gene Nup44A (FBgn0033247) is shown in Figures 2 and 3. Terms and definitions used in genetic modifier information pages are listed in Table 2.

**Table 2:** Terms used in NeuroGeM

**Terms**	**Values**	**Meaning**
Organism	*D. melanogaster*	Three model organisms
	*C. elegans*	
	*S. cerevisiae*	
Gene ID	FBgn------	Primary IDs used in the respective genome databases (FlyBase, WormBase, and SGD). These IDs are also used as primary IDs in NeuroGeM.
	W--------	
	S---------	
Type of modification	Suppressor	Suppressors are those genes that alleviate disease pathology or slow disease progression when over-expressed, and those that aggravate disease pathology or accelerate disease progression when down-regulated or deleted. Enhancers are those genes that alleviate disease pathology when down-regulated, and those that aggravate pathology when over-expressed. Non-modifiers have no effect on disease progression.
	Enhancer	
	Non-modifier	
Mode of action	Toxicity modification	Toxicity modifiers are those genes that change disease pathology. Aggregation modifiers are those genes that change the size or number of protein aggregates.
	Aggregation modification	
Disease model	Alzheimer’s disease (AD)	ND models compiled in the current version of NeuroGeM. We divided AD into the subtypes AD_Tau_ and AD_Aβ_ according to the gene used to induce the disease phenotype (mutant Tau protein and Aβ42, respectively).
	Huntington’s disease (HD)	
	Parkinson’s disease (PD)	
	Spinocerebellar ataxia type 1 (SCA1)	
	Spinocerebellar ataxia type 3 (SCA3)	
	Spinocerebellar ataxia type 7 (SCA7)	
	Amyotrophic lateral sclerosis (ALS)	
	PolyQ disease (PolyQ)	
Disease induction	Various	This field contains expression cassette information described in the literature including promoter and disease-causing gene (e.g. polyQ stretch length).
		Disease-causing mutant proteins compiled in NeuroGeM are Aβ and tau protein for AD, SOD1 for ALS, huntingtin for HD, α-synuclein for PD, Ataxin-1 for SCA1, Ataxin-3 (MJD) for SCA3, Ataxin-7 for SCA7 and polyQ stretches for the PolyQ disease model.
Modulation method	Overexpression	This field describes whether the expression level of the target gene increased (overexpression or gain-of-function) or decreased (knockdown, knockout, or loss-of-function). We adopted the same terms used in the original articles.
	Gain-of-function	
	Knockdown	
	Knockout	
	Loss-of-function	
Experimental scale	Primary high-throughput	This field describes the scale of the experiments. Experiments that were not high-throughput (**HT**) were assigned as low-throughput (**LT**). Experiments performed in primary screens were assigned as *Primary high-throughput*. Experiments to confirm the results obtained from the *Primary high-throughput* screens were assigned as *Secondary high-throughput*.
	Secondary high-throughput	
	Low-throughput	
Measurement	Various	This field describes how the change of pathology was evaluated. For example, change in the eye phenotype is a common readout in *D. melanogaster*, and cell growth rate is a common readout in *S. cerevisiae*.
Cell type	Various	This field briefly describes what cell lines and organs were utilized to carry out the experiment.

At the top of the genetic modifier information page (Figure 2a), the number of experimental records that are available in NeuroGeM for a specific gene and its homologs are reported (record summary) for each experimental scale (“L” stands for LT, “H_2_” for secondary HT, and “H_1_” for primary HT). If experimental data that identifies a specific gene as a modifier has been entered in NeuroGeM, this fact is highlighted in red, light red if it is only one HT experiment, and dark red if it is at least two HT experiments or at least one LT experiment. Similarly, records that report that a gene is a non-modifier are colored in light and dark blue. For example, there are currently two records in NeuroGeM for Nup44A indicating that Nup44A (FBgn0033247) is a non-modifier in HD, one based on a LT and one on a primary HT experiment. In addition, there are two records of LT experiments indicating that Nup44A is a modifier in SCA1. By clicking on a specific record in the “record summary” at the top of the page (e.g. “2 0 0” under the column header “L H_2_ H_1_” for Nup44A and SCA1), the user is immediately guided to the details of that entry (Figure 3).

Below the report summary, detailed information of the gene is displayed on the left side (Figure 2b), including synonyms, alternative gene names, and IDs used in other databases as well as GO annotations. On the right side (Figure 2c), NeuroGeM displays the protein-protein interaction sub-network around the gene, in which interacting proteins are colored by the type and result of experiments that tested them as modifiers. Specifically, the left half of each node (protein) is colored according to the evidence for it being a modifier. The right half of each node is colored according to the evidence for it being a non-modifier. The same coloring scheme as for the record summary is used. Importantly, the protein-protein interaction network and the coloring are organism- and disease-specific (coloring by different diseases can be selected below the network). Users can navigate to other genetic modifier information pages by clicking on the corresponding nodes. Figure 2c shows the protein-protein interaction sub-network of Nup44A colored according to the results of screens for modifiers of HD in *Drosophila*. A look at the network immediately reveals that Nup44A, Nup75, and Rae1 were identified as non-modifiers, while Nup107 was identified as modifier of HD in *Drosophila*. By contrast, SmB, SmD1, Cbp20, and Nup154 were identified as modifiers in some experiments but not in others.

In the next section of the genetic modifier information page, experimental details are displayed (Figure 3). Experiments are categorized by the disease model, and the number of experimental records for each disease model is shown with respect to experimental scale by using the same coloring scheme as for the report summary. The reported experimental details include: (i) *type of modification;* indicates whether the experiment found the gene to be a suppressor, enhancer, or non-modifier, (ii) *mode of action;* reports whether the queried gene modified aggregation size/number or changed disease symptoms, (iii) *disease induction;* denotes which (mutant) gene was used to cause disease symptoms, and shows the mutant gene and its expression cassette information, (iv) *modulation method;* denotes how the expression of the queried gene was modulated (e.g. over-expressed, knocked out, repressed by RNAi), (v) *experimental scale;* denotes the scale of performed experiments (LT, primary HT, secondary HT; secondary HT stands for experiments to confirm the results obtained from primary HT experiments), (vi) *measurement;* denotes what was quantified to identify a modifier and (vii) *cell type;* denotes the cell lines or stocks utilized in the experiment. For instance, Nup44A was tested as modifier in a *Drosophila* model of SCA1 that was created by expressing Ataxin-1 with a polyQ expansion of 82 (Figure 3). The impact of the overexpression of Nup44A on the disease model was quantified based on changes in the severity of an eye phenotype. Nup44A was categorized as a suppressor, indicating that the over-expression of the Nup44A gene alleviated the severity of the eye phenotype.

Below the experimental details of a specific gene, its homologous genes are displayed with genetic information, protein interaction sub-network, and experimental details if available.

### Search for orthologs of human and mouse genes

The current version of NeuroGeM does not contain any genetic modifiers in human and mouse (to be included at a later stage). Nevertheless, researchers studying genetic modifiers of NDs in the three model organisms are likely to be interested in the homologous genes of the modifiers in higher organisms such as human and mouse. Thus, we also integrated human genome information from the EMBL and HGNC databases and mouse genome information from the EMBL and MGI databases. The user can search for human and mouse genes using their gene names or EMBL IDs, and then obtain not only information on the queried genes but also information on their homologous genes in *D. melanogaster*, *C. elegans*, and *S. cerevisiae*. For example, *Drosophila*’s Nup44A gene and its homologous genes in other species are listed in Figure 2a and the information on those genes is displayed at the bottom of the Nup44A gene page (omitted in Figures 2 and 3). The user can also search for human and mouse genes homologous to Nup44A by their gene names (Seh1l and SEH1L) or EMBL IDs (ENSMUSG00000079614 and ENSG00000085415) in the unified search box.

### Applications of data in NeuroGeM

In order to demonstrate the broad applicability of NeuroGeM and how it can provide new understanding, we performed a variety of meta-analyses using the genetic modifiers data from NeuroGeM. In this section, the results of the meta-analyses are discussed briefly. The detailed methods, results and discussion for the meta-analyses are available in the Additional file 1. Due to the abundance of both HT and LT experimental data from *D. melanogaster*, mainly results obtained from the meta-analysis of genetic modifiers of *D. melanogaster* are presented here. Results from the analysis of genetic modifiers in other model organism as well as the comparison of modifiers in all three model organisms are available in Additional file 1.

**Figure 4:**
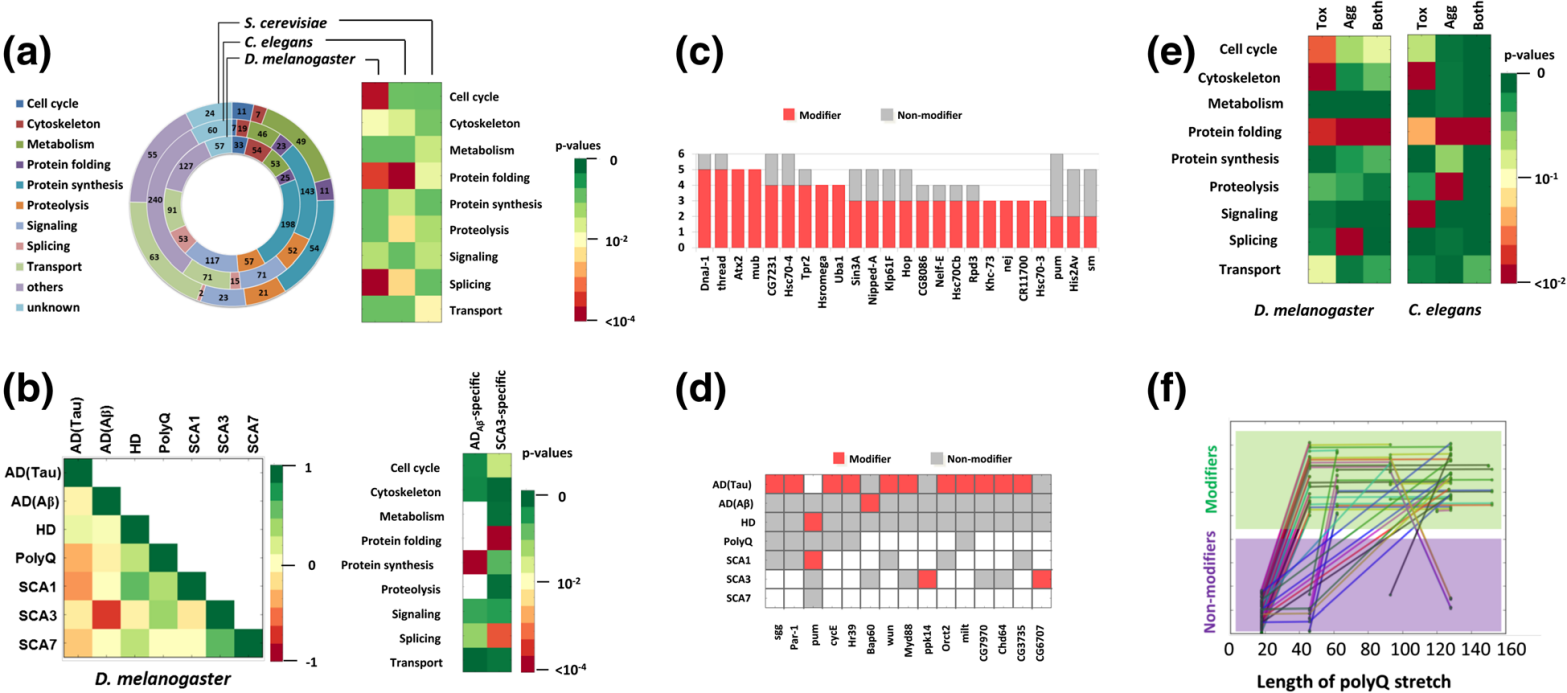
**Compilation of meta-analysis results. (a)** Functional classification of genetic modifiers and their enrichments represented by p-values. **(b)** (Left) Correlation analysis of modifiers and non-modifiers between diseases. Pairwise correlations (Matthew’s correlation coefficient) are represented by a color matrix ranging from −1 (inverse correlation, red), via 0 (no correlation, yellow), to 1 (high correlation, green). (Right) Modifiers in SCA3 and AD_Tau_ were further analyzed to see which functional categories are enriched among these genetic modifiers. **(c)** List of genes identified as modifiers of several diseases in *D. melanogaster*. Red and grey denote the number of diseases in which a gene is identified as a modifier and non-modifier, respectively (see Additional file 1: Figure S7 for full list). **(d)** List of disease-specific genetic modifiers. The same color annotation as in **(c)** is used here (see Additional file 1: Figure S8 for full list). **(e)** Enrichment analysis results of HD modifiers in *D. melanogaster* and PD modifiers in *C. elegans* with respect to the mode of action of the modifiers. Tox, toxicity modifiers; Agg, aggregation modifiers; Both, aggregation and toxicity modifiers. **(f)** Identification of modifiers and non-modifiers depending on the length of the polyQ stretch in HD models in *D. melanogaster*. Each line refers to one gene and each green dot refers to one experiment with a specific length of the polyQ stretch. If dots are in the purple and green region, it means the gene has been identified as a non-modifier and modifier, respectively. See Additional file 1 for details of meta-analysis.

i) NeuroGeM can be used to identify biological processes that are enriched within genetic modifiers in a specific disease or in groups of diseases. Genes with annotations for these processes can be prioritized for testing in other model organisms or for drug screenings. A meta-analysis of the data deposited in NeuroGeM revealed that modifiers across species are often involved in *protein folding* (Figure 4a). However, they account for only 3% of all genetic modifiers. The analysis revealed that modifiers are equally often involved in *cell cycle* and *splicing*, accounting for 7% and 3% of all genetic modifiers, respectively. This analysis result suggests that researchers expecting to discover more genetic modifiers should focus their efforts also on genes involved in *cell cycle* and *splicing*, biological processes that are also often enriched in modifiers. As shown in Figure 4b, a correlation analysis of modifiers between diseases revealed that polyQ diseases (HD, generic PolyQ, SCA1, SCA3, and SCA7 in Figure 4b) share many genetic modifiers and non-modifiers which are not seen in AD models, which is consistent with a previous report [21]. Specifically, a strong anti-correlation is observed when comparing the modifiers and non-modifiers of AD_Aβ_ and SCA3. Many SCA3-specific genetic modifiers are involved in *protein folding* and *splicing*[22,23], while AD_Aβ_-specific modifiers are involved in *protein synthesis*[24]*.* Untested genes involved in modifier-enriched processes can be prioritized in future screens to confirm these trends and elucidate their mechanisms. See Additional file 1.

ii) NeuroGeM allows easy identification (by non computational experts) of genes that modify the neurodegenerative toxicity in several ND models. Hence, cross-disease comparisons can identify potential *generic* modifiers that then can be tested experimentally in other disease models in other organisms (rodents) or compared to human genetic data. A first search for generic modifiers revealed that the genes DnaJ-1, thread, Atx2, and mub are modifiers in 5 out of 7 ND models in *D. melanogaster* (Figure 4c)*.* Interestingly, DNAJB4 and BIRC3, the mammalian orthologs of DnaJ-1 and thread*,* have recently been shown to reduce neuronal cell death when up-regulated in multiple mammalian ND models (see Additional file 1). Moreover, Atx2 has recently also been associated with an increased risk for ALS [25]. Further experiments are necessary to confirm this hypothesis that DnaJ-1, thread, Atx2, and mub are generic modifiers.

iii) Equally, NeuroGeM facilitates the identification of genes that only affect the phenotype of specific NDs. Though all NDs are caused by aggregates, they definitely show different pathophysiology. In this regard, genes capable of modulating disease phenotype only in a specific ND give us hints to understand the difference in disease progression. For instance, modifiers currently confined to AD_Tau_ in *D. melanogaster* include sgg and par-1 (Figure 4d). This finding is consistent with the specific importance of hyper-phosphorylation of the tau protein in AD, which is a process that may be accelerated by par-1 and sgg [26,27]. In *S. cerevisiae*, modifiers are enriched in *protein synthesis* in AD (RTG3, TEC1, SPT21, PPR1, MBP1, SRO9, SLF1, and SLS1), *protein folding* in HD (HSP26, HSP42 and APJ1), and *transport* in PD (FUN26, YCK3, and GOS1), which is consistent with recent results (Additional file 1).

iv) The database can help get new insights into the mechanism of disease modulation. In NeuroGeM, each modifier is classified as toxicity modifier and/or an aggregation modifier: toxicity modifiers change disease phenotype (eye development, motility, *etc.*), and aggregation modifiers primarily affect aggregate size or number. As shown in Figure 4e, toxicity modifiers are enriched in *cell cycle*, *cytoskeleton*, *signaling,* and *protein folding* categories; these modifiers are involved in cellular pathways that modulate the level of tolerance to the stress caused by the aggregates and ultimately lead to phenotypic changes. On the other hand, aggregation modifiers are enriched in *splicing, proteolysis,* and *protein folding*, which are the processes directly or indirectly associated with aggregate formation and elimination. Interestingly, modifiers belonging to both groups are only enriched in *protein folding*. This functional category includes protein quality control, which is a network of cellular processes that in an orchestrated manner works against protein misfolding and aggregation. Therefore, proteins involved in *protein folding* would be prime therapeutic targets, since they are able to resolve the problem of aggregate formation, and are involved in cellular processes that can increase the tolerance to the cellular stress caused by protein aggregation [28,29]. Moreover, these proteins may play a key role in the pathophysiology of many NDs due to their dual effect. In order to test this hypothesis, we identified *Drosophila* modifiers that are both aggregation and toxicity modifiers. We found that many of them are indeed able to modulate neurodegeneration in several different disease models (see Table 3 and Additional file 1). Interestingly, the list includes three of the previously identified generic modifiers; DnaJ-1, thread and Atx2. Next, we tested whether this is true across species, *i.e*. also for higher organisms. We identified human and mouse orthologs of the *Drosophila* aggregation and toxicity modifiers by using the feature of NeuroGeM. A careful literature search confirmed that for most of the mammalian orthologs of these modifiers there exists experimental evidence that they modify the phenotype of several ND models in mammalian cells (see Table 3 and Additional file 1 for details about the orthologs).

**Figure 5:**
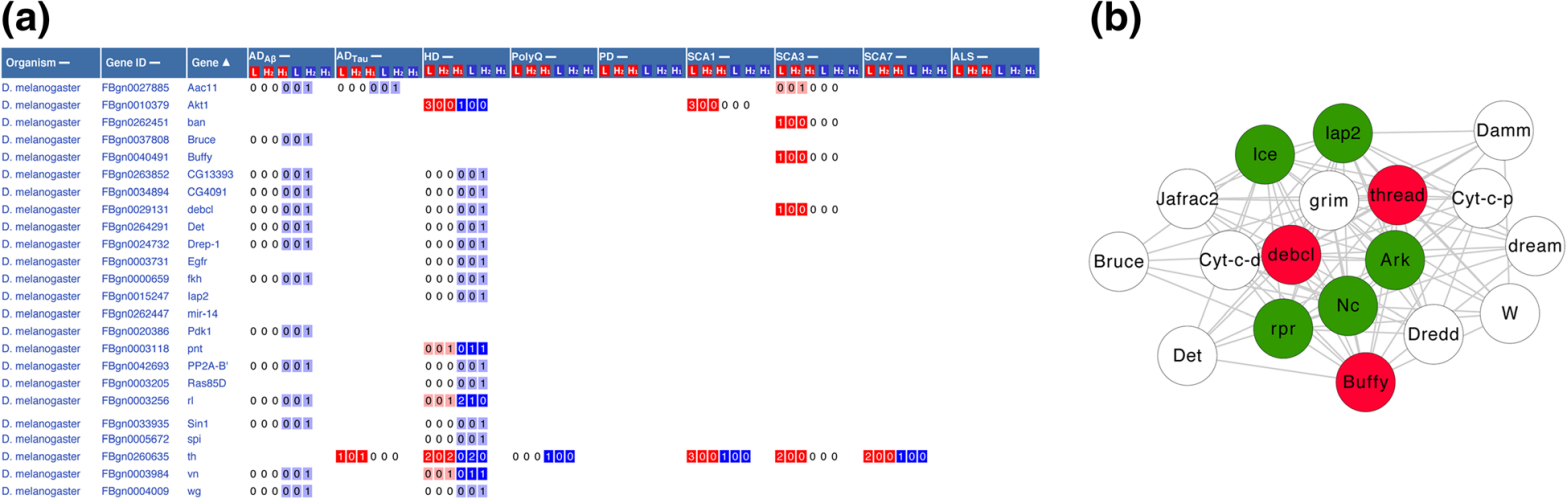
**Ontology-based search results of “*anti-apoptosis*”. (a)** Genes retrieved from NeuroGeM using the Ontology-based search for “anti-apoptosis” (GO:0006916). Genes annotated with anti-apoptosis or its child terms are listed. **(b)** Protein interaction clusters of the retrieved genes. Proteins that are directly interacting with the discovered modifiers are shown: red nodes, known modifiers; white nodes, untested genes; green nodes, genes with literature evidence (not yet entered into NeuroGeM) for being modifiers.

v) NeuroGeM allows assessing the effect of experimental conditions on the consistency and reliability of the identified modifiers. The results of different screens for genetic modifiers are often inconsistent because of the use of different experimental set-ups. NeuroGeM enables the user to infer the best experimental conditions for consistent identification of modifiers. As an example, we investigated the effect of polyQ stretch length on modifier identification in HD models of *D. melanogaster.* In Figure 4f, each line refers to one gene identified as a modifier or non-modifier in secondary HT or LT experiments in HD models with different polyQ lengths, and each green dot on the line refers to the identification result at a specific polyQ length. Figure 4f suggests some genes were not identified as modifiers in HD models with a polyQ length of 40 (which is above the canonical threshold of 35), but were then identified as modifier in models with a polyQ length of 60. Hence, this analysis suggests that HD models with polyQ > 60 may provide more sensitivity (see Additional file 1 for details).

vi) Most importantly, NeuroGeM facilitates the identification of new, so far untested modifiers. Mapping of genes on the protein interaction networks allows identification of new, untested genetic modifiers based on guilt-by-association. We illustrate this idea on genes involved in *anti-apoptosis* (GO:0006916). We first obtained 24 anti-apoptotic proteins in *D. melanogaster* (Figure 5a)*,* and among these genes debcl, Buffy, and thread are interconnected with each other in the protein network (Figure 5b). In order to investigate whether genes interacting with these *anti-apoptotic* modifiers could also be modifiers, we extended the sub-network by adding proteins that interact with the three proteins. This extension can be easily done, as NeuroGeM allows the user to navigate from one gene to another by clicking on a node in a network. The newly added genes are highly interconnected with each other. Detailed literature surveys of the genes connected to debcl, Buffy, and thread revealed that 5 out of 15 interactors (marked in green in Figure 5b) are modifiers or at least highly related to disease progression. For example, one of the interactors is Ark; inactivation of Ark (FBgn0263864), a key regulator of apoptosis, is known to suppress formation and ubiquitination of polyQ aggregates [107] (Please see Additional file 1 for the details about the other four interactors and potential modifiers). Hence, the search tools of NeuroGeM will facilitate the identification of new modifiers that are involved in specific pathways or cellular processes.

**Table 3:** **Genes that are toxicity and aggregation modifiers in *D. melanogaster *** **and their orthologs in *H. sapiens *** **and *M. musculus***^***1***^

***D. melanogaster *** **genes**	***D. melanogaster *** **ND models**^**2**^	**Ref**	***H. sapiens *** **orthologs**	***M. musculus *** **orthologs**	**Ref**^**3**^
DnaJ-1	AD_Tau_, HD, PolyQ, SCA1, SCA3	[30-34]	DNAJB1, DNAJB4, DNAJB5, DNAJB13	Dnajb1, Dnajb4, Dnajb5, Dnajb13	[35-40]
thread	AD_Tau_, HD, SCA1, SCA3, SCA7	[21,32,41,42]	BIRC2, BIRC3	Birc2, Birc3	[43-45]
Atx2	AD_Tau_, HD, SCA1, SCA3, SCA7	[21,41,42,46]	Atxn2, Atxn2L	Atxn2, Atxn2L	[47-52]
Hsc70-3	HD, SCA1, SCA7	[32,42]	HSPA5	Hspa5	[53]
Hsc70Cb	AD_Tau_, HD, SCA3	[5,54,55]	HSPH1(HSP110), HSPA4, HSPA4L	Hsph1 (Hsp110), Hspa4, Hsp4l	[56,57]
Rpd3	HD, SCA1, SCA7	[34,42,58]	HDAC1, HDAC2	Hdac1, Hdac2	[59-65]
14-3-3epsilon	HD, SCA1	[66,67]	YWHAZ, YWHAB, YWHAE	Ywhaz, Ywhab, Ywhae	[67-73]
CG5537	HD	[74]	UPRT	Uprt	N/A
Hsf	HD, SCA3	[75]	Hsf2, Hsf4, Hsfx1, Hsfx2, Hsfy1, Hsfy2, Hsf5	Hsf2, Hsf3, Hsf4, Hsfy2, Hsf5	[76-78]
Nipped-A	HD, SCA3, SCA7	[42,54,55]	TRRAP	Trrap	[79,80]
Sec61alpha	HD, SCA3	[81]	Sec61A1, Sec61A2	Sec61a1, Sec61a2	[82,83]
Nup160	HD, SCA3	[55,74]	Nup160	NUP160	[84-86]
CG1109	HD	[74]	WDR33(WDC146)	Wdr33 (Wdc146)	N/A
Snap	HD	[66]	NAPA, NAPB	Napa, Napb	N/A
smt3	HD	[74]	SUMO1, SUMO2, SUMO3, SUMO4	Sumo1, Sumo2, Sumo3	[87-91]
Mef2	HD	[66]	MEF2A, MEF2B, MEF2BNB, MEF2C, MEF2D	Mef2a, Mef2b, Mef2c, Mef2d	[92-97]
chic	HD	[98]	PFN4	Pfn4	[99]
Rpt1	HD	[74]	PSMC2	Psmc2	[100-102]
Sin3A	HD, SCA1, SCA3	[5,32,34]	Sin3A, Sin3B	Sin3a, Sin3b	[103]
Rheb	AD_Tau_, HD	[74,104]	Rheb, RhebL1	Rheb, Rhebl1	[105,106]

^1^Orthologs were obtained from NeuroGeM in which protein homolog groups of NCBI HomoloGene and InParanoid were integrated.

^2^Disease models in which the genes were identified as modifiers.

^3^Studies in which the mammalian orthologs were identified as modifiers.

## Conclusion

Here we report, to the best of our knowledge, the first database (NeuroGeM) of genetic modifiers of NDs. NeuroGeM provides a platform for searching modifiers, retrieving experimental conditions used for modifier identification, interpreting the roles of a queried modifier in the context of the protein interaction network, and expanding knowledge in one organism to other organisms through homologous genes. Therefore, NeuroGeM allows users to evaluate their hypotheses and develop new research directions. Furthermore, NeuroGeM provides all information, including gene information, protein interactions, experimental set-ups, *etc.* in down-loadable files, which will facilitate other computational analyses of modifiers similar to the meta-analysis presented in this work. Consequently, NeuroGeM will assist scientists immensely in their search for the pathophysiological mechanisms underlying NDs by providing the first compendium that catalogues and cross-links their genetic modifiers.

## Availability and requirements

NeuroGeM can be accessed from a web browser and is available at http://chibi.ubc.ca/neurogem.

## Abbreviations

ND: Neurodegenerative diseases; AD: Alzheimer’s disease; ADTau: Alzheimer’s disease caused by tau; ADAβ: Alzheimer’s disease caused by Aβ; ALS: Amyotrophic lateral sclerosis; PD: Parkinson’s disease; PolyQ: PolyQ disease; SCA1: Spinocerebellar ataxia type 1; SCA3: Spinocerebellar ataxia type 3; SCA7: Spinocerebellar ataxia type 7; HT: High-throughput; LT: Low-throughput; GO: GeneOntology.

## Competing interests

The authors declare that they have no competing interests.

## Authors’ contribution

DN built the database of genetic modifiers with integration of available databases and performed meta-analysis of genetic modifiers. DN, JG, CO, and DR designed the database and web site. MR implemented the web site to access the database of genetic modifiers. JG supervised the project. DN and JG wrote the manuscript. All authors read and approved the final manuscript.

## Pre-publication history

The pre-publication history for this paper can be accessed here:

http://www.biomedcentral.com/1755-8794/6/52/prepub

## Supplementary Material

Additional file 1 A text with figures addressing the meta-analysis results in detail mentioned in the main text. Click here for file

Additional file 2: Figure S1 A figure to illustrate a categorical search. Click here for file

Additional file 3: Figure S2 A figure to illustrate an ontology-based search. Click here for file
