# Supplementary material for: NeuroGeM, a knowledgebase of genetic modifiers in neurodegenerative diseases
Source: BMC Med Genomics. 2013 Nov 14;6:52. doi: 10.1186/1755-8794-6-52 (PMC3833180; doi:10.1186/1755-8794-6-52)
Supplement: Additional file 1 — A text with figures addressing the meta-analysis results in detail mentioned in the main text. [file 1755-8794-6-52-S1.docx]

**Supplementary Text**

**NeuroGeM, a knowledgebase of genetic modifiers in neurodegenerative diseases**

Dokyun Na, Mushfiqur Rouf, Cahir J. O’Kane, David C. Rubinsztein, and Jörg Gsponer

**Meta-analysis**

We performed a first meta-analysis of the data compiled in NeuroGeM. We identified cellular processes that are enriched with modifiers, compared genetic modifiers and non-modifiers between different NDs, identified modifiers that are common to groups of NDs or specific to some of them, extensively surveyed the literature to find links from the modifiers in the three model organisms to those in higher organisms, and inferred the effect of experimental conditions on the consistency of modifier identification.

***Identification of biological processes enriched among genetic modifiers***

The collected data of genetic modifiers allows us to identify relevant biological processes that are enriched within genetic modifiers, and genes in these processes can be prioritized for drug screens or for testing in other organisms. For this analysis, we categorized genetic modifiers according to their functional annotations in GeneOntology (GO), and then calculated the enrichment of each category using a *term*-for-*term* analysis based on a hypergeometric distribution [1] (Figure 4a). The analysis indicates that genes involved in *cell cycle*, *protein folding* and *splicing* are more likely to be genetic modifiers than those in other categories. Disease- and species-specific classifications are shown in Figure S3, S4 and S5. The enrichment of genes with annotations linked to *protein folding* is expected, because protein misfolding and aggregation is believed to play an essential role in the pathogenesis of NDs [2], and thus genes involved in protein quality control are likely to modify disease progression [3]. For this reason, the disease-modifying effect of heat shock proteins (HSP) has been widely studied in model organisms [4–7]. In addition to HSPs, transcription factors regulating the expression of HSPs have also been identified as modifiers [8]. Many studies have reported that HSPs can act as modifiers of different NDs in different model organisms [5, 9, 10]. Furthermore, the expression of genes encoding HSPs has been shown to be affected by toxic aggregates in ND models in mouse and human cells [11–13].

The enrichment for genes involved in *cell cycle* or *splicing* may appear more surprising. However, severe accumulation of aggregated proteins can trigger cellular stresses, and excessive stresses beyond the capacity of the cell will interrupt the cell cycle and induce cell death [14, 15]. Therefore, genes promoting cell division while suppressing apoptosis are likely to be modifiers not only in the model organisms [16, 17] but also in mammalian organisms [18].


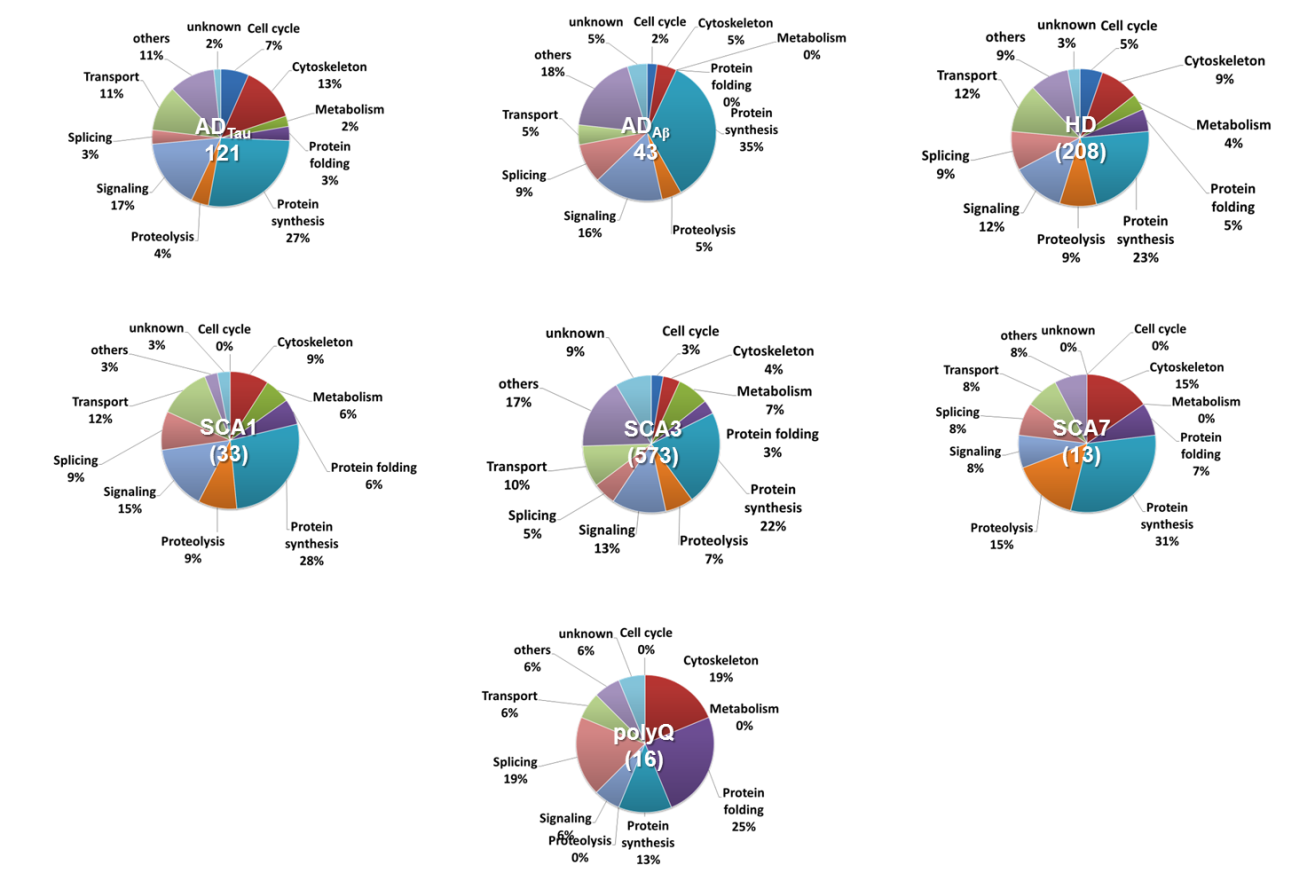


**Figure S3**. Classification of genetic modifiers in *D. melanogaster*

**
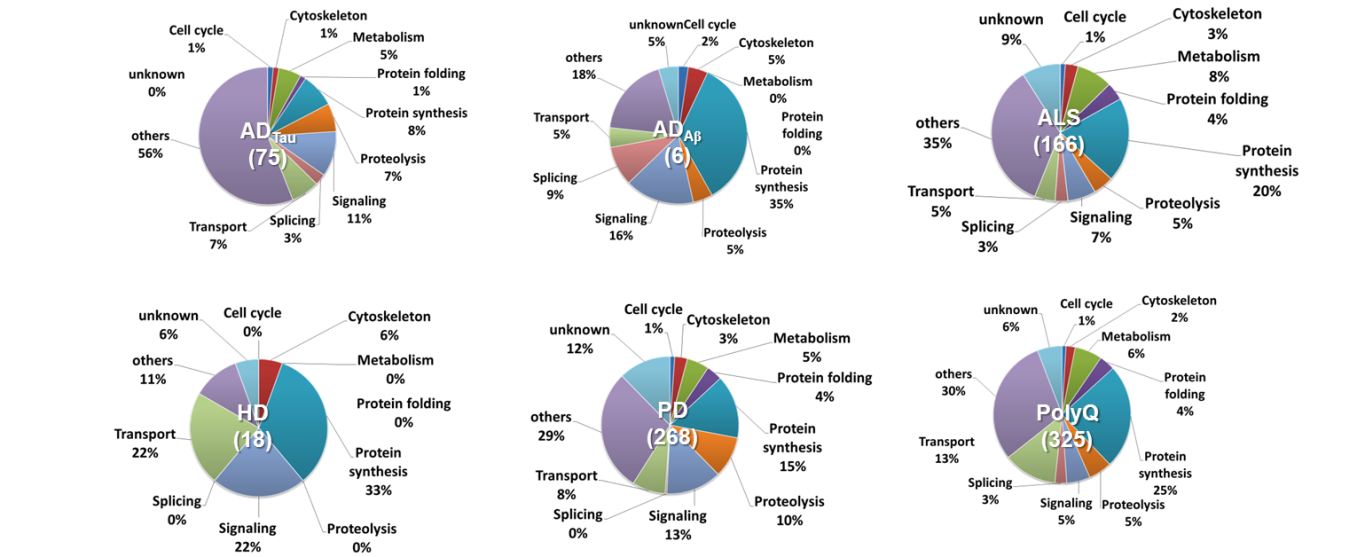
**

**Figure S4**. Classification of genetic modifiers in *C. elegans*


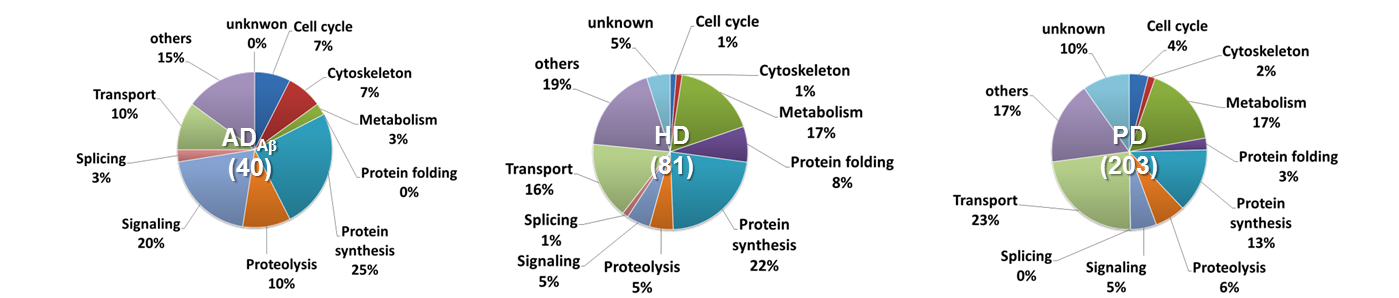


**Figure S5**. Classification of genetic modifiers in *S. cerevisiae*

***Correlation analysis of modifiers and non-modifiers between diseases***

Protein misfolding and aggregation are features common to NDs. Hence, one may expect that different NDs share at least some of the same modifiers. In order to investigate this hypothesis, we performed pairwise comparisons of diseases’ modifiers and non-modifiers. Genes that have been identified as either suppressors or enhancers at least once in a LT or HT experiment were regarded as modifiers. Any other tested genes were regarded as non-modifiers. This two-class categorization enabled us to apply well-established correlation-scoring methods. Due to the large bias towards non-modifiers, Mathew’s correlation coefficients (MCC) were calculated for the pairwise comparison (Figure 4b and Figure S6a-c). The MCC is defined as:


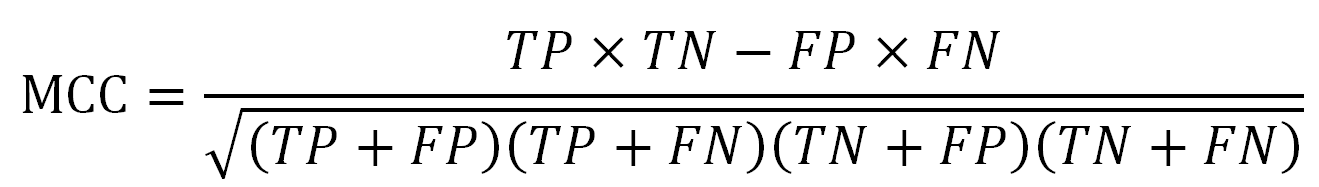


TP: Both genes are modifiers,
TN: Both genes are non-modifiers
FP/FN: One is a modifier and the other is a non-modifier.


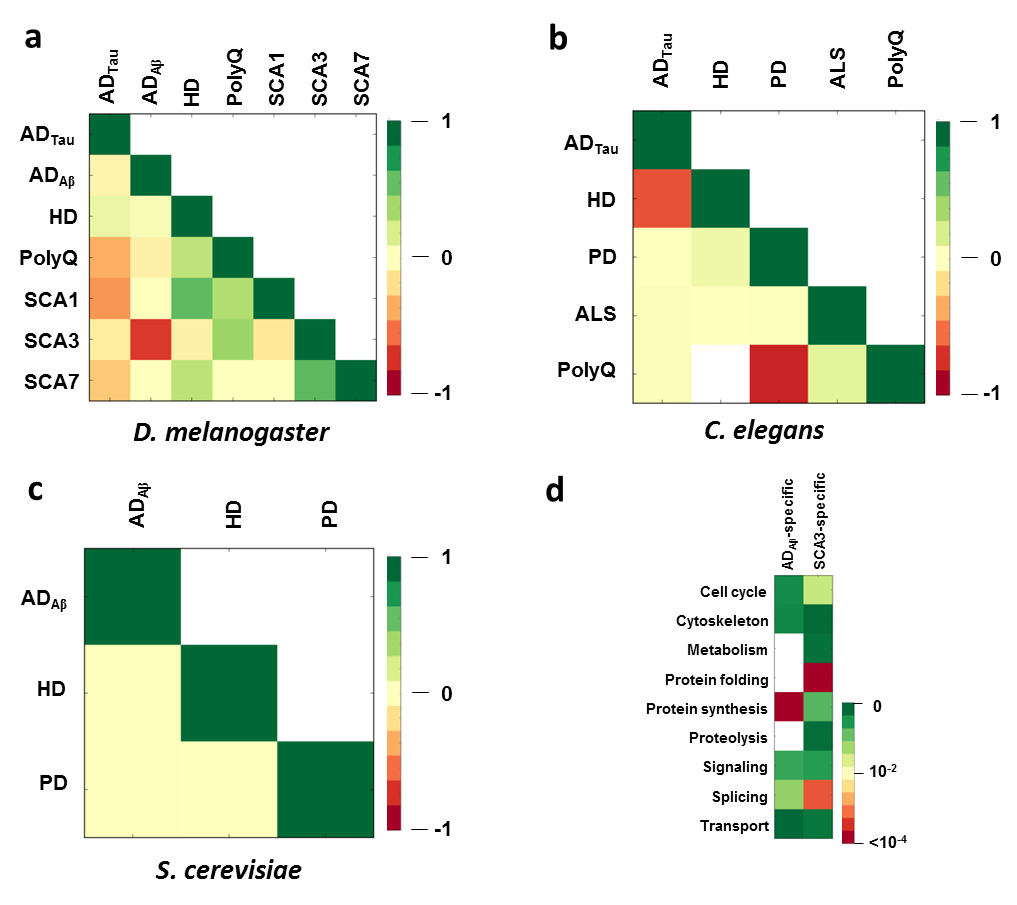


**Figure S6**. Modifier correlations across diseases. Pairwise correlation results (MCCs) of modifiers in *D. melanogaster* (**a**), *C. elegans* (**b**) and *S. cerevisiae* (**c**) are shown. (**d**) Functional categories enriched among modifiers and non-modifiers that are anti-correlated in AD_Aβ_ and SCA3 in *D. melanogaster*.

For *D. melanogaster*, this analysis revealed that, as expected [19], polyQ diseases (HD, SCA1, SCA3, SCA7, PolyQ) share a number of genetic modifiers and non-modifiers while they share far fewer modifiers and non-modifiers with AD. Indeed a strong anti-correlation is observed when comparing the modifiers and non-modifiers of AD_Aβ_ and SCA3. In order to gain further insight into this “anti-correlation”, we conducted an enrichment analysis of functional categories for genes that are modifiers in the AD_Aβ_ disease model but are not in the SCA3 model and *vice versa* (Figure S6d**).** Many SCA3-specific genetic modifiers are involved in *protein folding* (p-value of 10^-64^) and splicing (p-value of 4.5×10^-4^). In contrast, many genes involved in *protein synthesis* have been found to modify the phenotype in the AD_Aβ_ models (p-value of 1.52×10^-11^), but less so in SCA3 (p-value of 0.19). It is well established that chaperones modulate the neurotoxicity of polyglutamine aggregates and that their over-expression can suppress neurodegeneration in *Drosophila* and human cells [20, 21]. Recent studies also suggest that alternative splicing of the disease-causing protein in SCA3, Ataxin-3, may modulate neurotoxicity in mice [22, 23]**.** Support for the finding that genes involved in *protein synthesis* could be important modifiers in AD comes from recent experiments that show that the translation initiation factor eIF2α modulates the AD phenotype in mammalian disease models [24–26]. In any case, it has to be stressed that our correlation analysis of the data currently available in NeuroGeM does not indicate that genes involved in *protein synthesis* play no role in SCA3 and that those involved in *protein folding* and *splicing* play no role in AD. Our analysis just indicates that some genes involved in *protein folding* and *splicing* have been found to be modifiers in SCA3 but not in AD and *vice versa*. Similarly, the correlation analysis also reveals that modifiers and non-modifiers are more similar between SCA3 and SCA7 than between these two ataxias and SCA1, which has not been reported before. As the number of genes that could be used to calculate the MCC varies between diseases, the currently observed trends have to be confirmed when the coverage is more complete. Most importantly, this type of analysis, which identifies gene classes that are more likely to harbor modifiers of a specific disease, are now easily feasible thanks to NeuroGeM. Other genes with similar GO annotations can then be prioritized for future screens.

We conducted the same analysis for modifiers identified in *C. elegans* and *S. cerevisiae.* For *C. elegans*, the analysis shows negative correlation between modifiers and non-modifiers in HD and AD_Tau_, and PolyQ and PD, respectively (Figure S6b). The anti-correlation between modifiers and non-modifiers in HD and AD_Tau_ has to be interpreted with caution as the number of genes that could be used to calculate the MCC is small. No similar trends could be observed in *S. cerevisiae* because of the small overlap in identified modifiers in the different disease models (Figure S6c).

***Generic modifiers and disease specific modifiers***

The identification of modifiers that are shared between different NDs, as well as disease-specific modifiers, may provide important clues to pathophysiological processes that are generic to NDs or specific to some of them. Therefore, we searched first for genes that were identified as modifiers in several of the ND models. In *S. cerevisiae* only 5 genes (MUM2, YPL067C, STP2, TVP15 and HSP104) are modifiers that are shared by two different ND models. Genes that were identified as modifiers in more than one disease model in *D. melanogaster* and *C. elegans* are shown in Figure S7**.** Similar to *S. cerevisiae*, there are no genes in *C. elegans* that are modifiers in more than 3 disease models. In *D. melanogaster*, by contrast, DnaJ-1, thread, Atx2, and mub are modifiers in 5 out of 7 ND models (two subtypes of AD (Aβ and Tau), HD, SCA1, SCA3, SCA7, and PolyQ). DnaJ-1 is a heat shock protein, thread is an apoptotic suppressor, and Atx2 is a regulator of actin filament formation. The function of Mub is still unclear, but it is predicted to have a role in mRNA splicing. DnaJ-1 and thread are suppressors, meaning that elevating their activity alleviates toxic effects, while Atx2 is an enhancer. Mub is a suppressor in the AD_Tau_, SCA1, SCA3, and SCA7 models but is an enhancer in the HD model.

A careful literature survey confirmed that mammalian orthologs of these generic modifiers are also capable of modulating disease phenotypes in multiple NDs. In detail, the human ortholog of *Drosophila* DnaJ-1, DNAJB4 (ENSG00000162616), was found to reduce neuronal cell death when overexpressed in models of SCA1 [27, 28], SCA3 [29], Spinal and bulbar muscular atrophy (SBMA) [30], and HD [30, 31], and is associated with human PD [32]. BIRC3 (ENSMUSG00000032000), the mouse ortholog of thread, also rescues neuronal cell death when up-regulated by the overexpression of CREB in a mouse model of AD [33]. Human BIRC3 expression is down-regulated by Aβ [34]. Overexpression of BIRC3 helps neuronal cells survive by promoting anti-apoptotic activity; thus BIRC3 is expected to modulate neurodegeneration [35]. For Atx2, see ***Toxicity modifiers versus aggregation modifiers***.


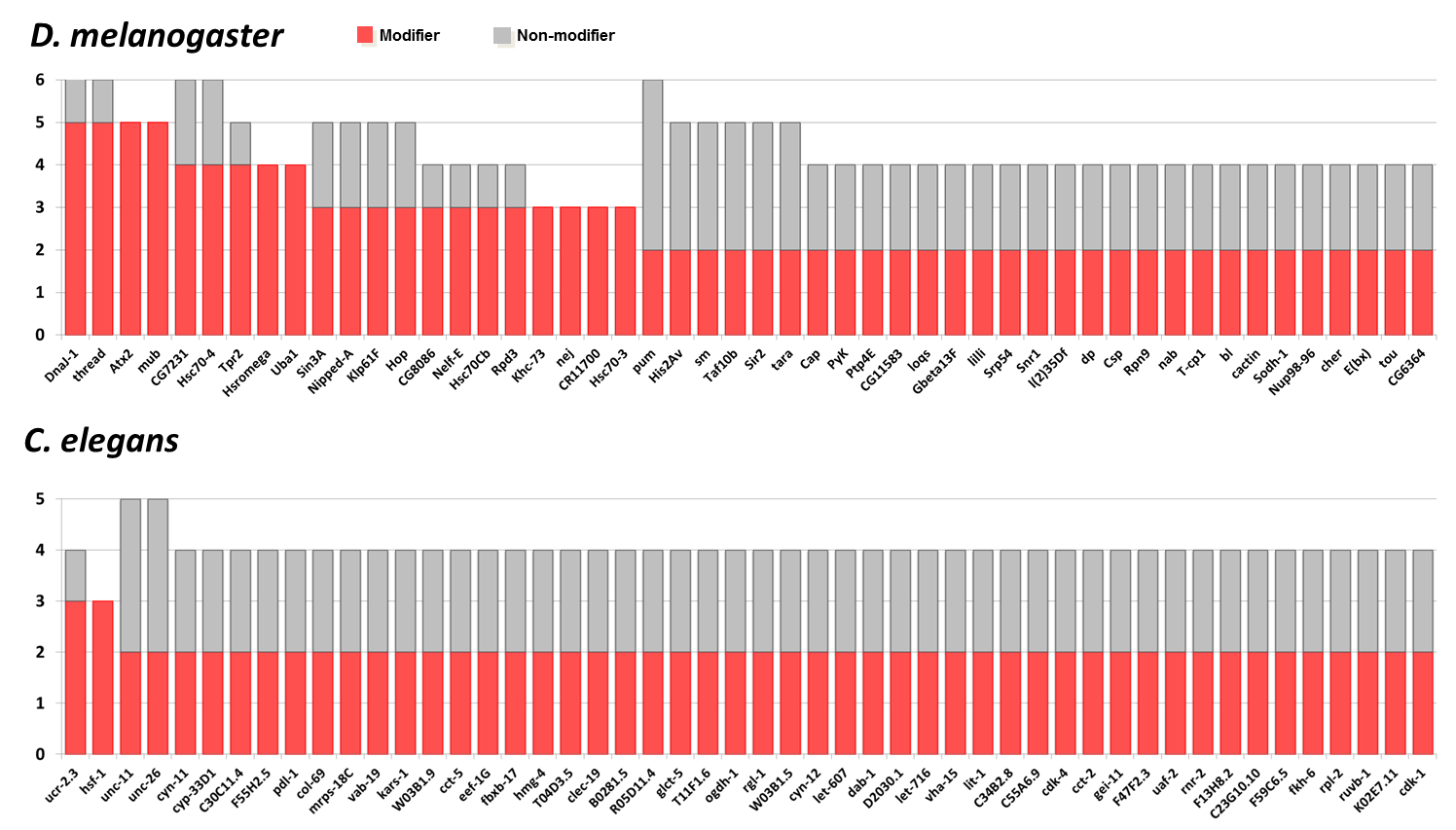


**Figure S7**. Number of diseases in which a specific gene is a modifier. Top 50 genes that affect several diseases are shown.

In contrast to generic modifiers, disease-specific modifiers could assist in the understanding of disease-specific mechanisms. We used order statistics to find disease-specific modifiers [36]. Genes examined in at least three different disease models were considered in the calculation and the top 50 disease-specific genes ordered by p-values are shown in Figure S8. In *D. melanogaster*, we find a large number of disease-specific modifiers for AD, specifically AD_Tau_. This finding may not be surprising given that AD is not caused by poly-Q expansions like HD, SCA1, SCA3 and SCA7, which are the other ND models in *Drosophila* with significant amounts of data. More interesting are the comparisons between AD, HD and PD in *S. cerevisiae.* Because most screens that have been carried out with this organism are HT in nature, nearly all *S. cerevisiae* genes have been tested as modifiers for AD, HD and PD. 260 genes were identified as modifiers in one of the three diseases but not in the others, i.e. they are predicted to be disease-specific. Consistent with the results in Figure S6d for *D. melanogaster*, genes related to *protein synthesis* are abundant among the AD-specific modifiers. These modifiers are involved in transcription (RTG3, TEC1, SPT21, PPR1, and MBP1) and translation (SRO9, SLF1, and SLS1). In the HD models, disease-specific modifiers are related to *protein folding*, which includes chaperones (HSP26, HSP42, and APJ1). In the PD models, disease-specific modifiers are often involved in vesicle transport (FUN26, YCK3, and GOS1). These findings are also consistent with recent results obtained from other species, which stress the importance of extensive modulation of transcription and translation processes in AD [24–26, 37], proteostasis in HD [31, 38, 39] and vesicle trafficking in PD [40, 41].


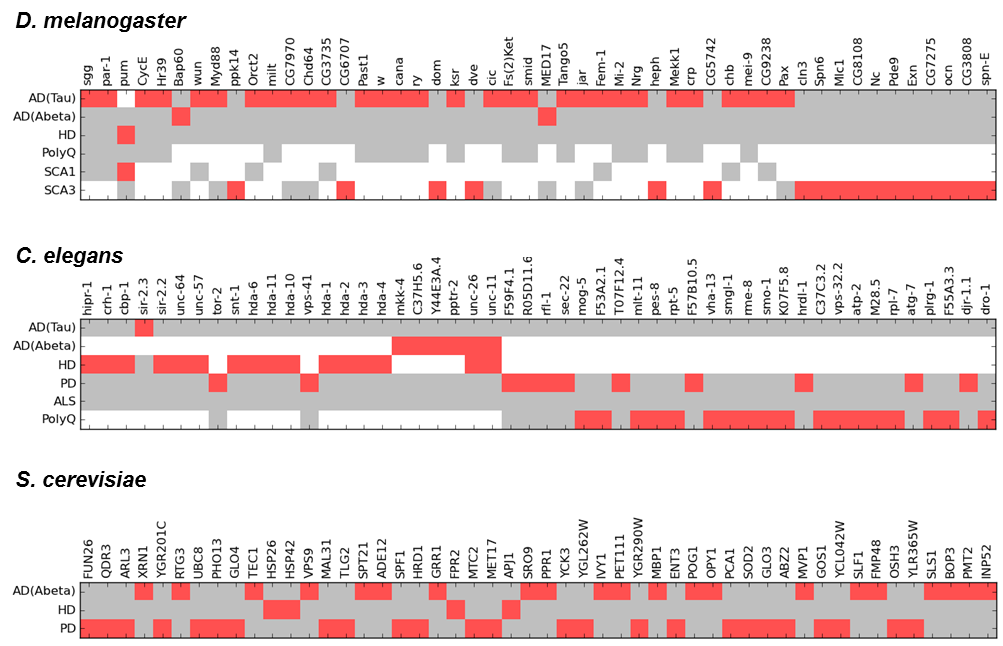


**Figure S8**. List of top 50 disease-specific genetic modifiers. Red and grey denote modifiers and non-modifiers, respectively. White denotes no available experimental data.

***Genetic modifiers conserved across species***

**Of particular interest are also genes that are found to be modifiers across species. Using the homolog information in NeuroGeM, we searched for groups of homologous genes whose members are modifiers in all the three model organisms (*D. melanogaster*, *C. elegans*, and *S. cerevisiae*). First, we looked for homologous genes that modify a specific disease in all three organisms. No such genes were found. Then, we looked for genes that are modifiers in all the three model organisms without distinction of disease model. We found 8 groups of homologous genes that modify at least one disease in all the three species (Table S1). These groups of homologous genes were identified by calculating *p*-value based on hypergeometric distribution from the occurrence of modifiers out of tested genes within a homolog group (p<0.001).**

**The identified groups of homologous genes are involved in very different biological processes (Table S1), ranging from transcription and translation over nuclear export to proteasome function and vesicle trafficking. Many genes that are associated with these functions have already been found previously to impact ND progression [24–26, 42]. However, our comparison of modifiers across species has to be interpreted with care because many of the genes in the 8 groups of homologs were tested only once as modifiers and were often only hits in primary HT screens.**

**Table S1. Genetic modifiers conserved across species.**

| **Function of ortholog group** | ***D. melanogaster*** | ***C. elegans*** | ***S. cerevisiae*** | ***M. musculus*** | ***H. sapiens*** |
| --- | --- | --- | --- | --- | --- |
| **ATPase of proteasome** | **Rpt5 (HD)** | **rpt-5 (PolyQ)** | **RPT5 (PD)** | **Psmc3** | **PSMC3** |
| **Transcription** | **Atms (SCA3)** | **C55A6.9 (PolyQ, ALS)** | **PAF1 (HD)** | **Paf1** | **Paf1** |
| **Vesicle trafficking** | **Rab1 (HD, PD)** | **rab-1 (PolyQ)** | **YPT1 (PD)** | **RAB1, RAB1B** | **RAB1A, RAB1B** |
| **Nuclear exporter** | **emb (AD_Tau_, SCA3)** | **xpo-1  (PolyQ, ALS)** | **CRM1 (AD_Aβ_)** | **XPO1** | **XPO1** |
| **Ribosome** | **RpL19 (HD)** | **rpl-19 (PolyQ)** | **RPL19B (PD)** | **RPL19** | **Rpl19** |
| **Casein kinase regulating vesicle fusion** | **gish (HD)** | **csnk-1 (PD)** | **YCK3 (PD)** | **Csnk1g1, Csnk1g2, Csnk1g3** | **CSNK1G1, CSNK1G2, CSNK1G3** |
| **Regulatory subunit of protein phosphatase 2A (PP2A)** | **wdb (AD_Tau_)** | **pptr-2 (AD_Aβ_)** | **RTS1 (AD_Aβ_)** | **Ppp2r5a, Ppp2r5b, Ppp2r5d, Ppp2r5e** | **PPP2R5A, PPP2R5C, PPP2R5D, PPP2R5E** |
| **Regulatory subunit of protein phosphatase Glc7p** | **CG9238 (AD_Tau_)** | **H18N23.2 (ALS)** | **GIP2 (PD)** | **Ppp1r3b, Ppp1r3c, Ppp1r3d** | **PPP1R3B, PPP1R3C, PPP1R3D** |

*** Genes from *D. melanogaster*, *C. elegans*, and *S. cerevisiae* were identified as genetic modifiers, while their orthologs in *M. musculus* and *H. sapiens* have not been tested yet.**

***Toxicity modifiers versus aggregation modifiers***

Modifiers can be grouped into aggregation modifiers and toxicity modifiers depending on the quantification method: the primary effect of aggregation modifiers is to increase or decrease aggregates while the primary effect of toxicity modifiers is to change the phenotype eventually leading to cell death. Investigating these two different types of modifiers is likely to provide important insight into two distinct, key steps of the pathophysiology of neurodegeneration.

We analyzed modifiers of the HD model in *D. melanogaster* and the PD model in *C. elegans*; they are chosen due to the abundance of aggregation and toxicity modifiers for both of these models. We found 77 toxicity modifiers and 151 aggregation modifiers for the HD model in *D. melanogaster*, and 68 toxicity modifiers and 204 aggregation modifiers for the PD model in *C. elegans*. These modifiers were then categorized according to their GO annotations into 9 categories and the statistical significance of each category was calculated. In the statistical test, all the evaluated genes were used as a reference set.

In the HD model in *D. melanogaster*, aggregation modifiers were enriched in *protein folding* and *splicing* while toxicity modifiers were enriched in *cell cycle*, *cytoskeleton*, and *protein folding* (Figure 4e). Interestingly, *protein folding* was the only category that was enriched within the modifiers that belong to both modifier groups. A very similar trend was observed in the PD models of *C. elegans*: *protein folding* was a commonly enriched category in both aggregation and toxicity modifiers. In addition, *signaling* was enriched among toxicity modifiers and *proteolysis* was enriched among aggregation modifiers. These results support the hypothesis that aggregation modifiers directly modulate the formation of aggregates while toxicity modifiers regulate cell tolerance against aggregate-induced stresses.

From the list of HD modifiers of *D. melanogaster*, we identified 20 genes that are both toxicity and aggregation modifiers (Table 3). Interestingly, modifiers that belong to the both groups included DnaJ-1, thread and Atx2. These modifiers were found to be generic modifiers in our meta-analysis, which means that they modulate neuronal death in multiple ND models. Likewise, many other modifiers belonging to both groups are modifiers in more than one disease model in *D. melanogaster*. These results suggest that modifiers capable of both controlling aggregation formation and regulating cell tolerance to aggregates could play a key role in the pathophysiology of many NDs.

To test this hypothesis, we verified whether homologs of genes that are aggregation and toxicity modifiers in ND models in *D. melanogaster* are also modifiers in mammalian systems. Hence, we searched for mammalian orthologous genes of the 20 aggregation and toxicity modifiers (Table 3) by using NeuroGeM. A careful literature search confirmed that there exists experimental evidence that most of the mammalian orthologs can modify several mammalian ND models. In the following, we discuss details of these mammalian homologs:

- DNAJB4 and BIRC3 are orthologous genes of the generic modifiers, DnaJ-1 and thread of *Drosophila,* respectively, and their abilities to modulate neurodegenerative toxicity were already summarized in the section, ‘***Generic modifiers and disease specific modifiers***’.

- Atxn2 is an orthologous gene of *Drosophila*’s Atx2 that is also a generic modifier. In higher organisms, the polyQ extension within Atxn2 causes a neurodegenerative disorder, SCA2 [43], and Atxn2 is thought to produce toxic effects by forming aggregates [44]. Thus, Atxn2 is commonly utilized to build SCA2 models [44]. In human, Atxn2 and TDP-43 were highly colocalized in ALS patients [45], and recent studies revealed that Atxn2 with an intermediate length of polyQ (27-33) is associated to ALS [45–48].

- HSPA5 is an ortholog of *Drosophila*’s Hsc70-3, a member of Hsp70 family. The expression of the chaperone protein HSPA5 was reduced in a mouse model of Spinocerebellar ataxia type 17 [49]. In this model the disease-causing mutant protein, TBP, tightly binds to the transcription factor nuclear factor-Y and prevents the transcription factor from initiating the transcription of chaperone genes including HSPA5 and Hsp70. Shortly, the mutant TBP reduces the expression level of HSPA5, and thereby reduces the level of cellular response to stress. Thus, up-regulation of HSPA5 is expected to alleviate the neurodegenerative toxicity.

**- HSPH1 (HSP110) is an orthologous gene of *Drosophila*’s Hsc70Cb (HSP110, dHSP110), a member of Hsp70 family. Recently, HSPH1 has been reported to function as a nucleotide exchange factor for Hsp70 chaperones and constitute an additional component of Hsp70 machinery [50]. Mice with deletion of the HSPH1 gene (-/-) exhibit accumulation of hyperphosphorylated tau and insoluble amyloid beta (Aβ42) [51], leading to AD. In addition, deletion of HSPH1 leads to a similar phenotype as the deletion of Hsp70, which is a potent suppressor in multiple species [51, 52]. Over-expression of human HSPH1 suppresses cell death as well as aggregation formation in cell-based SBMA models [53]. Thus, HSPH1 is capable of modulating neurotoxicity.**

- HDAC1 and HDAC2 are othologs of *Drosophila*’s histone acetylase, Rpd3. The level of histone deacetylases (HDACs) in mouse HD models was correlated with disease progression [54], and inhibition of HDACs alleviates neurodegenerative symptoms in HD models [54–58], the ALS model [59], and the AD model [60].

- 14-3-3 proteins (YWHAZ, YWHAB, YWHAE) are orthologous genes of *Drosophila*’s 14-3-3epsilon, a positive regulator of the Ras-mediated signaling pathway. They are known to be associated with many different NDs [61–65]. Specifically, a high level of plasma homocysteine (Hcy) increases the risk of developing NDs such as AD. Hcy is known to down-regulate the YWHAE gene in rat hippocampal neurons in a dose-dependent manner, inducing neuronal apoptosis [66]. The YWHAZ gene is known to facilitate the formation of aggregates and its repression by using siRNA suppresses aggregate formation in a cell-based animal HD model [67].

- Hsf2 and Hsf4 are orthologs of *Drosophila*’s Hsf. They are members of many heat shock proteins that are transcriptionally regulated by a master heat shock factor, Hsf1 [68]. Loss of the Hsf2 gene increases the accumulation of aggregates and shortens the life span of HD mice [68], and Hsf2 was associated with mutant SOD-1 induced ALS [69]. In another report, loss of either Hsf2 or Hsf4 exacerbated the progressive myelin loss of mice [70].

- TRRAP is an orthologous gene of *Drosophila*’s Nipped-A, a member of Tip60 chromatin-remodeling complex involved in DNA repair. Atxn7 is known to function in the chromatin remodeling complexes of TFTC (GCN5 and TRRAP) and STAGA [SPT-TAF(II)31-GCN5L acetylase], and polyQ-extension of Atxn7 disrupts the function of these complexes and causes SCA7 [71, 72].

- **SEC61A1 and A2 are orthologous genes of *Drosophila*’s Sec61alpha and components of the SEC61 complex. The ER-associated degradation process (ERAD) ensures that misfolded polypeptides are retro-translocated to the cytosol for proteasomal degradation. The SEC61 complex is involved in the translocation of polypeptides across the ER membrane. Ataxin-3 is a p97-associated deubiquitinating enzyme mutated in SCA3 and defects in its function result in failure in ERAD. Thus, ataxin-3 is likely to contribute to the mechanisms that lead to the translocation of misfolded proteins to the cytosol [73, 74]. As SEC61A1 and A2 are also involved in the translocation across ER to cytosolic proteasomes, they could be implicated in SCA3. Consistent with this idea, up-regulation of SEC61alpha in a *Drosophila* SCA3 model alleviates disease severity [75].**

- NUP160 is an ortholog of *Drosophila*’s Nup160. NUP160 serves as a scaffold component of nuclear pore complexes. Interestingly, the life-span of NUP160 is 2-3 years [76], and thus NUP160 can be damaged due to exposure to age-related toxic metabolites. Malfunctional NUP160 leads to an increased accumulation of cytosolic proteins inside the nucleus, i.e., accumulation of tubulin aggregates in old rat brains [42, 77]. These results imply the potential association of NUP160 with ND [42].

- SUMO proteins are orthologous genes of *Drosophila*’s smt3. They are small ubiquitin-like modifiers that modify proteins post-translationally. It has been reported that several pathogenic polyQ proteins for HD, SCA1, SCA7, SBMA, *etc* are post-translationally modified by SUMO proteins [78–80]. It was also found that decreasing SUMO activity by the mutation of the Ataxin-7 SUMO site in a mouse SCA7 model increased insoluble aggregates that are toxic to the cell [78]. Therefore, SUMO proteins would function as suppressors. Along with these results, enhancement of ubiquitination activity by over-expressing ubiquitin ligase genes reduces polyQ aggregates in mammalian cell-based models [81] and decrease of ubiquitination activity accelerates neuropathology [82].

- MEF2 proteins are orthologs of *Drosophila*’s Mef2. Many isoforms belong to this myocyte enhancer factor-2 group (MEF2). They are transcription factors that enhance neuronal survival. Their expression level is reduced in PD patients and a rat PD model [83]. In a cell-based mouse PD model, disruption of MEF2s impaired neuronal cell viability [84] while promotion of MEF2 activity protected neuronal cells from death [85]. Furthermore, MEF2A, a member of MEF2 group, is known to be associated with increased risk of developing AD [86, 87].

- PFN4 is an orthologous gene of *Drosophila*’s chic that affects cytoskeleton structure. The PFN protein has four isoforms. They bind to actin monomers to regulate cytoskeleton formation. As PFN is up-regulated in PD patients and change in neurofilaments takes place during the progression of PD, PFN is believed to be one of the factors affecting neurodegenerative symptoms [88].

- PSMC2 is an orthologous gene of *Drosophila*’s Rpt1. PSMC2 protein is a member of 26S proteasome. PSMC5 is a proteasome inhibitor that sequesters PSMC2 to prevent the formation of 26S proteasome. According to previous reports, proteasome inhibition causes the formation of aggregation and mice with overexpression of PSMC5 show aging-associated phenotypes [89–91]. Therefore, the PSMC5’s target protein, PSMC2, is likely to be associated with neurodegenerative phenotypes.

- Sin3A is an ortholog of *Drosophila*’s Sin3A. Sin3A is a transcriptional repressor when in complex with HDAC, coREST, REST, and other proteins. This complex prevents the expression of brain-derived neurotrophic factor (BDNF). Several studies have reported that in patients with AD, PD, and HD, the mRNA and protein levels of BDNF were reduced, and overexpression of BDNF in mice improved neurophysiology [92]. Thus, it is believed that the complex harboring Sin3A is associated with ND. In addition, wild-type human Htt protein sequesters REST in the cytoplasm and thereby prevents the formation of the complex. On the contrary, the polyQ-expanded Htt protein fails to capture the REST protein, and as a result the transcription of the BDNF gene is repressed by the complex. Therefore, Sin3A is believed to be implicated in HD and other ND [92].

- Rheb (Ras homolog enriched in brain) is an ortholog of *Drosophila*’s Rheb. This protein regulates cell proliferation and cell cycle *via* the mTOR pathway, and also enhances apoptosis in response to stress [93, 94]. Rheb inhibits autophagy by activating the mTOR signaling pathway that negatively regulates autophagy. Consistent with this knowledge, over-expression of a constitutively active mutant form of human Rheb in mouse made axons of dopaminergic neurons resistant to retrograde degeneration [94].

Overall, we found ample literature evidence that supports our hypothesis that genetic modifiers capable of modulating aggregation formation and disease phenotypes may act as genetic modifiers across ND models and species, and such *generic* modifiers are likely to play an important role in the progression of NDs.

***Inference of the best experimental conditions for reliable and consistent modifier identification***

The identification of genetic modifiers of NDs is difficult due to the complex mechanisms that underlie these diseases. The experimental identification of genetic modifiers consists of several steps: (1) induction of a disease phenotype by (over)expressing one or several disease-causing genes, (2) modulation of the expression of a potential modifier gene, and (3) observation and quantification of the change in disease phenotype. In each of these three steps, many parameters have to be considered: (i) which disease-causing gene is expressed in which organ (eye, brain, or elsewhere; *cell type* in NeuroGeM) and at which level of severity (severe or mild; *disease induction* in NeuroGeM), (ii) how is the expression of the potential modifier changed (overexpressed, knocked down, or knocked out; *modulation method* in NeuroGeM), (iii) at which scale can the experiment be carried out (primary HT, secondary HT and LT; *experimental scale* in NeuroGeM) and (iv) how much change in the symptom(s) is required to identify a modifier (*measurement* in NeuroGeM). Due to this complexity, it is obvious that the identification of genetic modifiers of NDs is difficult and can lead to inconsistencies when results are generated in different conditions. Indeed, it is known that inconsistencies can result from off-target effects in RNAi screens, inconsistent knockdown in RNAi experiments (leading to false-negative results in some cases where no effects are observed), or due to the effect the tested genes have on the expression of the disease-causing gene itself [95, 96]. Moreover, knockdowns can affect dominant or recessive alleles resulting in different experimental readouts. Hence, the comparison of modifiers that were identified under different experimental conditions is very difficult. NeuroGeM provides the ideal framework to approach this difficult problem.

As a test case, we investigated the effect of polyQ stretch length on modifier identification in HD models in *D. melanogaster*. The results of the analysis are shown in Figure 4f. Each line refers to one gene identified as a modifier or non-modifier in secondary HT or LT experiments in HD models with different polyQ lengths, and each green dot on the line refers to the identification result at a specific polyQ length. For instance, the line for gene *mef2* (FBgn0011656) connects a first green dot in the non-modifier region at a polyQ length of 18 with a second dot in the modifier region at a polyQ length of 128. This line indicates that the first experiment was performed with polyQ=18 and identified the gene as a non-modifier, while the second experiment identified the gene as a modifier using polyQ=128. Figure 4f suggests that all of the target genes tested in a HD model with a polyQ length of 18 were identified as non-modifiers, while at a polyQ length larger than 60, most of them were identified as modifiers. Interestingly, some genes were not identified as modifiers in HD models with a polyQ length of 40 (which is above the canonical threshold of 35), but were then identified as modifier in models with a polyQ length of 60. Hence, HD models with polyQ>60 may provide more sensitivity.

***Identification of new, so far untested modifiers***

If several genes in the same cellular process have been identified as modifiers in ND models, it is likely that other genes in the same process and interacting with genetic modifiers could be modifiers as well. Here, by using NeuroGeM we examined genes involved in *anti-apoptosis* (GO:0006916) and investigated the hypothesis that proteins interacting with modifiers involved in *anti-apoptosis* are modifiers too.

Selecting “*Search in* *Ontologies”* and “*D. melanogaster*”, and entering “*anti-apoptosis”* or “GO:0006916” in the search box returns 24 *Drosophila* genes that have an annotation for *anti-apoptosis* or its child GO terms (Figure 5a). Due to high false positive rates of primary HT screens, we focused on results obtained from secondary HT and LT experiments. Of the 24 genes, 8 genes have been investigated in secondary HT or LT experiments: FBgn0010379 (Akt1), FBgn0260635 (thread, th), FBgn0029131 (debcl), FBgn0040491 (Buffy), FBgn0262451 (ban), FBgn0003984 (vein, vn), FBgn0003118 (pnt) and FBgn0003256 (rolled, rl).

Among these genes, debcl, Buffy, and thread are all modifiers in a *Drosophila* model of SCA3 and are interconnected with each other in a protein network (Figure 5b). In order to investigate whether genes interacting with these *anti-apoptotic* modifiers could also be modifiers, we extended the sub-network by adding proteins that interact with the three proteins. This extension can be easily done, as NeuroGeM allows the user to navigate from one gene to another by clicking on a node in a network. The newly added genes are highly interconnected each other and many of them are regulators of the three previously identified modifiers. As no experimental data for the newly added genes in LT and secondary HT are yet available in our database, they are good examples for where further hypothesis testing may be valuable. Detailed literature surveys of the genes connected to debcl, Buffy, and thread revealed that 5 out of 15 interactors (marked in green in Figure 5b) are modifiers or at least highly related to disease progression.

1. **Ark** (FBgn0263864)**:** Inactivation of Ark, an apoptosis regulator, inhibits formation of polyQ aggregates, and Ark is co-localized with ubiquitinated aggregates in *Drosophila*. These suggest that Ark plays a role in the formation of pathogenic polyQ-containing aggregates [97]. In addition, reducing the cellular level of Ark by over-expressing TRPC1, a negative regulator of Ark, inhibits degeneration of human neuroblastoma cells [98].
2. **rpr** (FBgn0011706)**:** rpr is known to regulate the strong modifier thread by promoting its degradation. Alteration of the activity of rpr is expected to modulate neuronal toxicity in *Drosophila* [99].
3. **Iap2** (FBgn0015247): Iap2 is a protein inhibitor of apoptosis (IAP). IAPs are overexpressed in many human malignancies, and the expression of IAP proteins in human AD and ALS are significantly altered [100]. Similarly, IAP proteins involved in the same apoptosis process in *Drosophila* are also expected to modulate neuronal degeneration.
4. **Nc** (FBgn0026404) and **Ice** (FBgn0019972): Nc and Ice are involved in the activation cascade of caspases responsible for apoptosis execution and are expected to modulate neurodegenerative diseases by regulating cell death. These proteins have been identified as modifiers in a primary HT screen of a SCA3 model in *D. melanogaster* [101] .

Similar to these experimentally tested genes, many of the other genes in the “*anti-apoptosis*” sub-network are highly likely to be modifiers as well.

**Data Upload**

**It is essential to continually update NeuroGeM and, thereby, provide up-to-date information to users. Basically, we frequently update NeuroGeM, but also allow users to submit their own data upon request of a login. Once submitted, the data is curated and will be released on NeuroGeM.**

**There is a clickable button at the bottom of our NeuroGeM front page, named ‘Upload Data’ (Figure S9a). After clicking this button, the user will be asked for a login ID and password, which will be provided by us upon request. After login, the user will see 5 buttons to upload files (Figure S9b). Our system is designed in such a way that it is able to process formatted files with large numbers of genetic modifiers, because more and more data is coming from high-throughput screening experiments. The user can upload not only genetic modifiers (‘Experiment file’) but also genetic information on the modifiers (‘Gene file’), GeneOntology information (‘Gene ontology file’), homologous gene data (‘Homologous gene file’), and protein-protein interaction (‘Gene interaction file’) data. File formats are identical to the file formats used on the download page (http://chibi.ubc.ca/neurogem/download.php). As an example, the file format for uploading genetic modifiers (‘Experiment file’) is shown in Figure S9c. The first row has column names that will be ignored when processing the file. In the second row, genetic modifier information including gene ID, disease model, experimental conditions, and modifier identification results should be entered. Details on file formats and column meanings are also available on the NeuroGeM help page.**


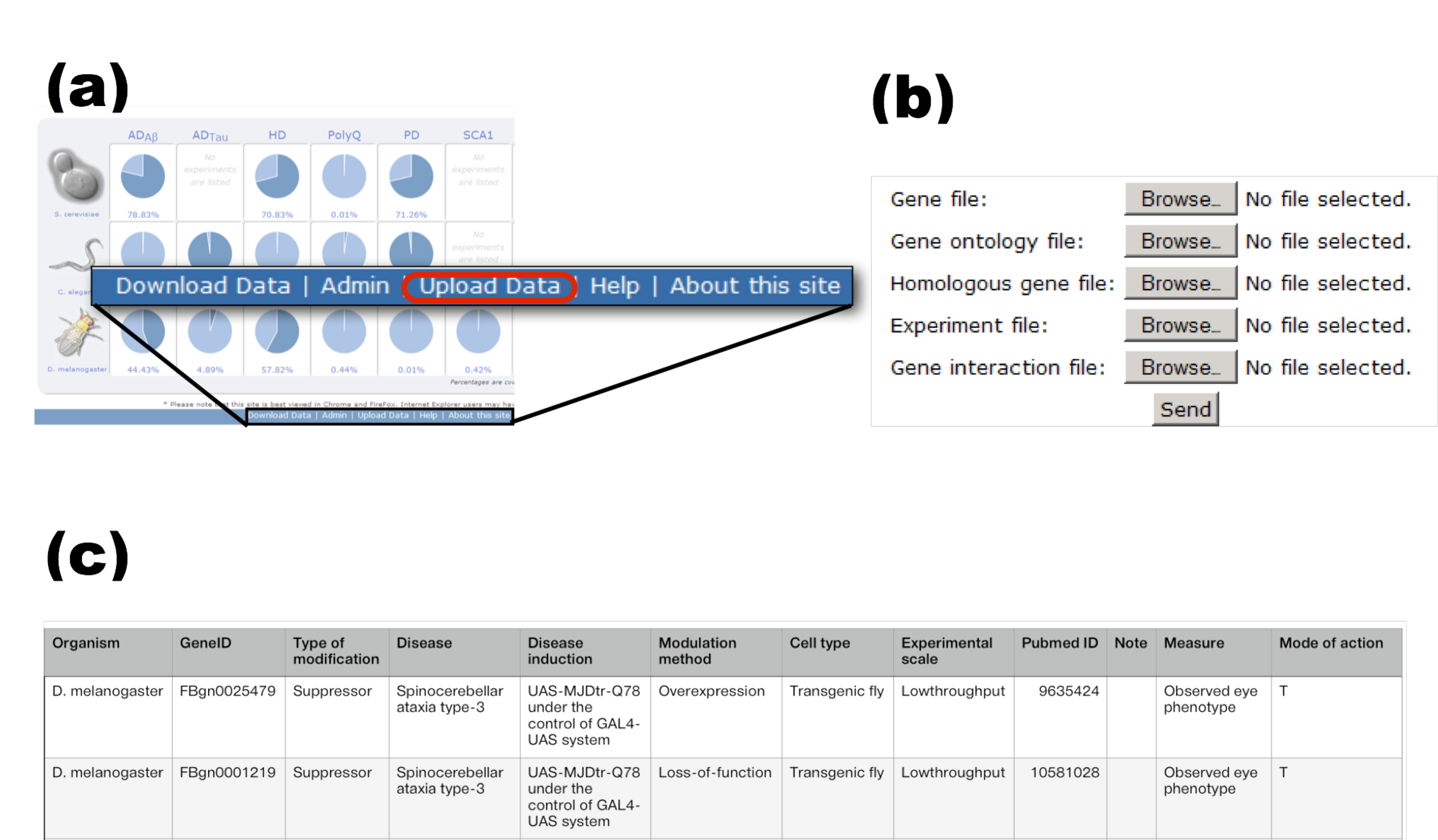


**Figure S9. Uploading user data**

**(a) A hyperlink to an upload page is available at the bottom of the NeuroGeM front page, named ‘Upload Data’. (b) Once logged in, five buttons to upload data will appear. Detailed information on the different files to upload, including their formats, is available on the NeuroGeM help page. (c) This figure shows the file format that has to be used for uploading results from genetic modifier screens.**

**References**

1. Grossmann S, Bauer S, Robinson PN, Vingron M: **Improved detection of overrepresentation of Gene-Ontology annotations with parent–child analysis**. *Bioinformatics* 2007, **23**:3024–3031.

2. Warrick JM, Paulson HL, Gray-Board GL, Bui QT, Fischbeck KH, Pittman RN, Bonini NM: **Expanded polyglutamine protein forms nuclear inclusions and causes neural degeneration in *Drosophila***. *Cell* 1998, **93**:939–949.

3. Morimoto RI: **The heat shock response: Systems biology of proteotoxic stress in aging and disease**. *Cold Spring Harb Symp Quant Biol* 2012, **76**:91–99.

4. Cashikar AG, Duennwald M, Lindquist SL: **A chaperone pathway in protein disaggregation**. *J Biol Chem* 2005, **280**:23869–23875.

5. Kazemi-Esfarjani P, Benzer S: **Genetic suppression of polyglutamine toxicity in *Drosophila***. *Science* 2000, **287**:1837–1840.

6. Carra S, Boncoraglio A, Kanon B, Brunsting JF, Minoia M, Rana A, Vos MJ, Seidel K, Sibon OCM, Kampinga HH: **Identification of the *Drosophila* ortholog of HSPB8**. *J Biol Chem* 2010, **285**:37811–37822.

7. Liao P-C, Lin H-Y, Yuh C-H, Yu L-K, Wang H-D: **The effect of neuronal expression of heat shock proteins 26 and 27 on lifespan, neurodegeneration, and apoptosis in *Drosophila***. *Biochem Bioph Res Co* 2008, **376**:637–641.

8. Fujikake N, Nagai Y, Popiel HA, Okamoto Y, Yamaguchi M, Toda T: **Heat shock transcription factor 1-activating compounds suppress polyglutamine-induced neurodegeneration through induction of multiple molecular chaperones**. *J Biol Chem* 2008, **283**:26188–26197.

9. Satyal SH, Schmidt E, Kitagawa K, Sondheimer N, Lindquist S, Kramer JM, Morimoto RI: **Polyglutamine aggregates alter protein folding homeostasis in *Caenorhabditis elegans***. *Proc Natl Acad Sci* 2000, **97**:5750–5755.

10. Shorter J: **Hsp104: A weapon to combat diverse neurodegenerative disorders**. *Neurosignals* 2008, **16**:63–74.

11. Cowan KJ, Diamond MI, Welch WJ: **Polyglutamine protein aggregation and toxicity are linked to the cellular stress response**. *Hum Mol Genet* 2003, **12**:1377–1391.

12. Hay DG, Sathasivam K, Tobaben S, Stahl B, Marber M, Mestril R, Mahal A, Smith DL, Woodman B, Bates GP: **Progressive decrease in chaperone protein levels in a mouse model of Huntington’s disease and induction of stress proteins as a therapeutic approach**. *Hum Mol Genet* 2004, **13**:1389–1405.

13. Zabel C, Chamrad DC, Priller J, Woodman B, Meyer HE, Bates GP, Klose J: **Alterations in the mouse and human proteome caused by Huntington’s disease**. *Mol Cell Proteomics* 2002, **1**:366–375.

14. Cooper AA, Gitler AD, Cashikar A, Haynes CM, Hill KJ, Bhullar B, Liu K, Xu K, Strathearn KE, Liu F, Cao S, Caldwell KA, Caldwell GA, Marsischky G, Kolodner RD, LaBaer J, Rochet J-C, Bonini NM, Lindquist S: **α-Synuclein blocks ER-Golgi traffic and Rab1 rescues neuron loss in Parkinson’s models**. *Science* 2006, **313**:324–328.

15. Lindholm D, Wootz H, Korhonen L: **ER stress and neurodegenerative diseases**. *Cell Death Differ* 2006, **13**:385–392.

16. Senoo-Matsuda N, Igaki T, Miura M: **Bax-like protein Drob-1 protects neurons from expanded polyglutamine-induced toxicity in *Drosophila***. *EMBO J* 2005, **24**:2700–2713.

17. Mattson MP: **Apoptosis in neurodegenerative disorders**. *Nat Rev Mol Cell Biol* 2000, **1**:120–129.

18. Friedlander RM: **Apoptosis and caspases in neurodegenerative diseases**. *N Engl J Med* 2003, **348**:1365–1375.

19. Shulman JM, Feany MB: **Genetic modifiers of tauopathy in *Drosophila***. *Genetics* 2003, **165**:1233–1242.

20. Gao X-C, Zhou C-J, Zhou Z-R, Zhang Y-H, Zheng X-M, Song A-X, Hu H-Y: **Co-chaperone HSJ1a dually regulates the proteasomal degradation of Ataxin-3**. *PLoS ONE* 2011, **6**:e19763.

21. Warrick JM, Chan HYE, Gray-Board GL, Chai Y, Paulson HL, Bonini NM: **Suppression of polyglutamine-mediated neurodegeneration in *Drosophila* by the molecular chaperone HSP70**. *Nat Genet* 1999, **23**:425–428.

22. Harris GM, Dodelzon K, Gong L, Gonzalez-Alegre P, Paulson HL: **Splice isoforms of the polyglutamine disease protein Ataxin-3 exhibit similar enzymatic yet different aggregation properties**. *PLoS ONE* 2010, **5**:e13695.

23. Switonski PM, Fiszer A, Kazmierska K, Kurpisz M, Krzyzosiak WJ, Figiel M: **Mouse ataxin-3 functional knock-out model**. *Neuromolecular Med* 2011, **13**:54–65.

24. O’Connor T, Sadleir KR, Maus E, Velliquette RA, Zhao J, Cole SL, Eimer WA, Hitt B, Bembinster LA, Lammich S, Lichtenthaler SF, Hébert SS, De Strooper B, Haass C, Bennett DA, Vassar R: **Phosphorylation of the translation initiation factor eIF2α increases BACE1 levels and promotes amyloidogenesis**. *Neuron* 2008, **60**:988–1009.

25. Gu X, Sun J, Li S, Wu X, Li L: **Oxidative stress induces DNA demethylation and histone acetylation in SH-SY5Y cells: potential epigenetic mechanisms in gene transcription in Aβ production**. *Neurobiol Aging* 2013, **34**:1069–1079.

26. Segev Y, Michaelson DM, Rosenblum K: **ApoE ε4 is associated with eIF2α phosphorylation and impaired learning in young mice**. *Neurobiol Aging* 2013, **34**:863–872.

27. Petrakis S, Raskó T, Russ J, Friedrich RP, Stroedicke M, Riechers S-P, Muehlenberg K, Möller A, Reinhardt A, Vinayagam A, Schaefer MH, Boutros M, Tricoire H, Andrade-Navarro MA, Wanker EE: **Identification of human proteins that modify misfolding and proteotoxicity of pathogenic Ataxin-1**. *PLoS Genet* 2012, **8**:e1002897.

28. Cummings CJ, Mancini MA, Antalffy B, DeFranco DB, Orr HT, Zoghbi HY: **Chaperone suppression of aggregation and altered subcellular proteasome localization imply protein misfolding in SCA1**. *Nat Genet* 1998, **19**:148–154.

29. Chai Y, Koppenhafer SL, Bonini NM, Paulson HL: **Analysis of the role of heat shock protein (Hsp) molecular chaperones in polyglutamine disease**. *J Neurosci* 1999, **19**:10338–10347.

30. Kobayashi Y, Kume A, Li M, Doyu M, Hata M, Ohtsuka K, Sobue G: **Chaperones Hsp70 and Hsp40 suppress aggregate formation and apoptosis in cultured neuronal cells expressing truncated androgen receptor protein with expanded polyglutamine tract**. *J Biol Chem* 2000, **275**:8772–8778.

31. Jana NR, Tanaka M, Wang G, Nukina N: **Polyglutamine length-dependent interaction of Hsp40 and Hsp70 family chaperones with truncated N-terminal huntingtin: their role in suppression of aggregation and cellular toxicity**. *Hum Mol Genet* 2000, **9**:2009–2018.

32. Long P, Samnakay P, Jenner P, Rose S: **A yeast two-hybrid screen reveals that osteopontin associates with MAP1A and MAP1B in addition to other proteins linked to microtubule stability, apoptosis and protein degradation in the human brain**. *Eur J Neurosci* 2012, **36**:2733–2742.

33. Pugazhenthi S, Wang M, Pham S, Sze C-I, Eckman CB: **Downregulation of CREB expression in Alzheimer’s brain and in Aβ-treated rat hippocampal neurons**. *Mol Neurodegener* 2011, **6**:60.

34. Valerio A, Boroni F, Benarese M, Sarnico I, Ghisi V, Bresciani LG, Ferrario M, Borsani G, Spano P, Pizzi M: **NF-κB pathway: a target for preventing β-amyloid (Aβ)-induced neuronal damage and Aβ42 production**. *Eur J Neurosci* 2006, **23**:1711–1720.

35. Knight JC, Scharf EL, Mao-Draayer Y: **Fas activation increases neural progenitor cell survival**. *J Neurosci Res* 2010, **88**:746–757.

36. Aerts S, Lambrechts D, Maity S, Van Loo P, Coessens B, De Smet F, Tranchevent L-C, De Moor B, Marynen P, Hassan B, Carmeliet P, Moreau Y: **Gene prioritization through genomic data fusion**. *Nat Biotech* 2006, **24**:537–544.

37. Ferrer I: **Differential expression of phosphorylated translation initiation factor 2 alpha in Alzheimer’s disease and Creutzfeldt–Jakob’s disease**. *Neuropathol Appl Neurobiol* 2002, **28**:441–451.

38. Gidalevitz T, Ben-Zvi A, Ho KH, Brignull HR, Morimoto RI: **Progressive disruption of cellular protein folding in models of polyglutamine diseases**. *Science* 2006, **311**:1471–1474.

39. Lotz GP, Legleiter J, Aron R, Mitchell EJ, Huang S-Y, Ng C, Glabe C, Thompson LM, Muchowski PJ: **Hsp70 and Hsp40 functionally interact with soluble mutant Huntingtin oligomers in a classic ATP-dependent reaction cycle**. *J Biol Chem* 2010, **285**:38183–38193.

40. Burré J, Sharma M, Tsetsenis T, Buchman V, Etherton MR, Südhof TC: **α-Synuclein promotes SNARE-complex assembly *in vivo* and *in vitro***. *Science* 2010, **329**:1663–1667.

41. Van Ham TJ, Thijssen KL, Breitling R, Hofstra RMW, Plasterk RHA, Nollen EAA: ***C. elegans* model identifies genetic modifiers of α-synuclein inclusion formation during aging**. *PLoS Genet* 2008, **4**:e1000027.

42. Toyama BH, Hetzer MW: **Protein homeostasis: live long, won’t prosper**. *Nat Rev Mol Cell Biol* 2013, **14**:55–61.

43. Kasumu AW, Hougaard C, Rode F, Jacobsen TA, Sabatier JM, Eriksen BL, Strøbæk D, Liang X, Egorova P, Vorontsova D, Christophersen P, Rønn LCB, Bezprozvanny I: **Selective positive modulator of calcium-activated potassium channels exerts beneficial effects in a mouse model of Spinocerebellar Ataxia Type 2**. *Chem Biol* 2012, **19**:1340–1353.

44. Damrath E, Heck MV, Gispert S, Azizov M, Nowock J, Seifried C, Rüb U, Walter M, Auburger G: **ATXN2-CAG42 sequesters PABPC1 into insolubility and induces FBXW8 in cerebellum of old ataxic knock-in mice**. *PLoS Genet* 2012, **8**:e1002920.

45. Elden AC, Kim H-J, Hart MP, Chen-Plotkin AS, Johnson BS, Fang X, Armakola M, Geser F, Greene R, Lu MM, Padmanabhan A, Clay-Falcone D, McCluskey L, Elman L, Juhr D, Gruber PJ, Rüb U, Auburger G, Trojanowski JQ, Lee VM-Y, Deerlin VMV, Bonini NM, Gitler AD: **Ataxin-2 intermediate-length polyglutamine expansions are associated with increased risk for ALS**. *Nature* 2010, **466**:1069–1075.

46. Nielsen TT, Svenstrup K, Budtz-Jørgensen E, Eiberg H, Hasholt L, Nielsen JE: **ATXN2 with intermediate-length CAG/CAA repeats does not seem to be a risk factor in hereditary spastic paraplegia**. *J Neurol Sci* 2012, **321**:100–102.

47. Lee T, Li YR, Ingre C, Weber M, Grehl T, Gredal O, Carvalho M de, Meyer T, Tysnes O-B, Auburger G, Gispert S, Bonini NM, Andersen PM, Gitler AD: **Ataxin-2 intermediate-length polyglutamine expansions in European ALS patients**. *Hum Mol Genet* 2011, **20**:1697–1700.

48. Ross OA, Rutherford NJ, Baker M, Soto-Ortolaza AI, Carrasquillo MM, DeJesus-Hernandez M, Adamson J, Li M, Volkening K, Finger E, Seeley WW, Hatanpaa KJ, Lomen-Hoerth C, Kertesz A, Bigio EH, Lippa C, Woodruff BK, Knopman DS, White CL, Gerpen JAV, Meschia JF, Mackenzie IR, Boylan K, Boeve BF, Miller BL, Strong MJ, Uitti RJ, Younkin SG, Graff-Radford NR, Petersen RC, et al.: **Ataxin-2 repeat-length variation and neurodegeneration**. *Hum Mol Genet* 2011, **20**:3207–3212.

49. Huang S, Ling JJ, Yang S, Li X-J, Li S: **Neuronal expression of TATA box-binding protein containing expanded polyglutamine in knock-in mice reduces chaperone protein response by impairing the function of nuclear factor-Y transcription factor**. *Brain J Neurol* 2011, **134**(Pt 7):1943–1958.

50. Dragovic Z, Broadley SA, Shomura Y, Bracher A, Hartl FU: **Molecular chaperones of the Hsp110 family act as nucleotide exchange factors of Hsp70s**. *EMBO J* 2006, **25**:2519–2528.

51. Eroglu B, Moskophidis D, Mivechi NF: **Loss of Hsp110 leads to age-dependent tau hyperphosphorylation and early accumulation of insoluble amyloid beta**. *Mol Cell Biol* 2010, **30**:4626–4643.

52. Quan S, Bardwell JCA: **Chaperone discovery**. *BioEssays* 2012, **34**:973–981.

53. Ishihara K, Yamagishi N, Saito Y, Adachi H, Kobayashi Y, Sobue G, Ohtsuka K, Hatayama T: **Hsp105α suppresses the aggregation of truncated androgen receptor with expanded CAG repeats and cell toxicity**. *J Biol Chem* 2003, **278**:25143–25150.

54. Quinti L, Chopra V, Rotili D, Valente S, Amore A, Franci G, Meade S, Valenza M, Altucci L, Maxwell MM, Cattaneo E, Hersch S, Mai A, Kazantsev A: **Evaluation of histone deacetylases as drug targets in Huntington’s disease models.** *PLoS Curr* 2010, **2**.

55. Thomas EA, Coppola G, Desplats PA, Tang B, Soragni E, Burnett R, Gao F, Fitzgerald KM, Borok JF, Herman D, Geschwind DH, Gottesfeld JM: **The HDAC inhibitor 4b ameliorates the disease phenotype and transcriptional abnormalities in Huntington’s disease transgenic mice**. *Proc Natl Acad Sci U S A* 2008, **105**:15564–15569.

56. Hockly E, Richon VM, Woodman B, Smith DL, Zhou X, Rosa E, Sathasivam K, Ghazi-Noori S, Mahal A, Lowden PAS, Steffan JS, Marsh JL, Thompson LM, Lewis CM, Marks PA, Bates GP: **Suberoylanilide hydroxamic acid, a histone deacetylase inhibitor, ameliorates motor deficits in a mouse model of Huntington’s disease**. *Proc Natl Acad Sci* 2003, **100**:2041–2046.

57. Ferrante RJ, Kubilus JK, Lee J, Ryu H, Beesen A, Zucker B, Smith K, Kowall NW, Ratan RR, Luthi-Carter R, Hersch SM: **Histone deacetylase inhibition by sodium butyrate chemotherapy ameliorates the neurodegenerative phenotype in Huntington’s disease mice**. *J Neurosci* 2003, **23**:9418–9427.

58. Chou CJ, Herman D, Gottesfeld JM: **Pimelic diphenylamide 106 is a slow, tight-binding inhibitor of class I histone deacetylases**. *J Biol Chem* 2008, **283**:35402–35409.

59. Ryu H, Smith K, Camelo SI, Carreras I, Lee J, Iglesias AH, Dangond F, Cormier KA, Cudkowicz ME, H. Brown R, Ferrante RJ: **Sodium phenylbutyrate prolongs survival and regulates expression of anti-apoptotic genes in transgenic amyotrophic lateral sclerosis mice**. *J Neurochem* 2005, **93**:1087–1098.

60. Kilgore M, Miller CA, Fass DM, Hennig KM, Haggarty SJ, Sweatt JD, Rumbaugh G: **Inhibitors of class 1 histone deacetylases reverse contextual memory deficits in a mouse model of Alzheimer’s disease**. *Neuropsychopharmacolog* 2009, **35**:870–880.

61. Chen H-K, Fernandez-Funez P, Acevedo SF, Lam YC, Kaytor MD, Fernandez MH, Aitken A, Skoulakis EMC, Orr HT, Botas J, Zoghbi HY: **Interaction of Akt-phosphorylated Ataxin-1 with 14-3-3 mediates neurodegeneration in Spinocerebellar ataxia type 1**. *Cell* 2003, **113**:457–468.

62. Kaneko K, Hachiya NS: **The alternative role of 14-3-3 zeta as a sweeper of misfolded proteins in disease conditions**. *Med Hypotheses* 2006, **67**:169–171.

63. Okamoto Y, Shirakashi Y, Ihara M, Urushitani M, Oono M, Kawamoto Y, Yamashita H, Shimohama S, Kato S, Hirano A, Tomimoto H, Ito H, Takahashi R: **Colocalization of 14-3-3 proteins with SOD1 in Lewy body-like hyaline inclusions in familial amyotrophic lateral sclerosis cases and the animal model**. *PLoS ONE* 2011, **6**:e20427.

64. Hashiguchi M, Sobue K, Paudel HK: **14-3-3ζ is an effector of tau protein phosphorylation**. *J Biol Chem* 2000, **275**:25247–25254.

65. Waelter S, Boeddrich A, Lurz R, Scherzinger E, Lueder G, Lehrach H, Wanker EE: **Accumulation of mutant huntingtin fragments in aggresome-like inclusion bodies as a result of insufficient protein degradation**. *Mol Biol Cell* 2001, **12**:1393–1407.

66. Wang J, Bai X, Chen Y, Zhao Y, Liu X: **Homocysteine induces apoptosis of rat hippocampal neurons by inhibiting 14-3-3ε expression and activating calcineurin**. *PLoS ONE* 2012, **7**:e48247.

67. Omi K, Hachiya NS, Tanaka M, Tokunaga K, Kaneko K: **14-3-3zeta is indispensable for aggregate formation of polyglutamine-expanded huntingtin protein**. *Neurosci Lett* 2008, **431**:45–50.

68. Shinkawa T, Tan K, Fujimoto M, Hayashida N, Yamamoto K, Takaki E, Takii R, Prakasam R, Inouye S, Mezger V, Nakai A: **Heat shock factor 2 is required for maintaining proteostasis against febrile-range thermal stress and polyglutamine aggregation**. *Mol Biol Cell* 2011, **22**:3571–3583.

69. Batulan Z, Shinder GA, Minotti S, He BP, Doroudchi MM, Nalbantoglu J, Strong MJ, Durham HD: **High threshold for induction of the stress response in motor neurons is associated with failure to activate HSF1**. *J Neurosci* 2003, **23**:5789–5798.

70. Homma S, Jin X, Wang G, Tu N, Min J, Yanasak N, Mivechi NF: **Demyelination, astrogliosis, and accumulation of ubiquitinated proteins, hallmarks of CNS disease in hsf1-deficient mice**. *J Neurosci* 2007, **27**:7974–7986.

71. Mookerjee S, Papanikolaou T, Guyenet SJ, Sampath V, Lin A, Vitelli C, DeGiacomo F, Sopher BL, Chen SF, Spada ARL, Ellerby LM: **Posttranslational modification of Ataxin-7 at lysine 257 prevents autophagy-mediated turnover of an N-Terminal caspase-7 cleavage fragment**. *J Neurosci* 2009, **29**:15134–15144.

72. Helmlinger D, Hardy S, Abou-Sleymane G, Eberlin A, Bowman AB, Gansmüller A, Picaud S, Zoghbi HY, Trottier Y, Tora L, Devys D: **Glutamine-expanded Ataxin-7 alters TFTC/STAGA recruitment and chromatin structure leading to photoreceptor dysfunction**. *PLoS Biol* 2006, **4**:e67.

73. Meusser B, Hirsch C, Jarosch E, Sommer T: **ERAD: the long road to destruction**. *Nat Cell Biol* 2005, **7**:766–772.

74. Wang Q, Li L, Ye Y: **Regulation of retrotranslocation by p97-associated deubiquitinating enzyme ataxin-3**. *J Cell Biol* 2006, **174**:963–971.

75. Kanuka H, Kuranaga E, Hiratou T, Igaki T, Nelson B, Okano H, Miura M: **Cytosol-endoplasmic reticulum interplay by Sec61α translocon in polyglutamine-mediated neurotoxicity in Drosophila**. *Proc Natl Acad Sci* 2003, **100**:11723–11728.

76. Savas JN, Toyama BH, Xu T, Yates JR, Hetzer MW: **Extremely long-lived nuclear pore proteins in the rat brain**. *Science* 2012, **335**:942–942.

77. D’Angelo MA, Raices M, Panowski SH, Hetzer MW: **Age-dependent deterioration of nuclear pore complexes causes a loss of nuclear integrity in postmitotic cells**. *Cell* 2009, **136**:284–295.

78. Janer A, Werner A, Takahashi-Fujigasaki J, Daret A, Fujigasaki H, Takada K, Duyckaerts C, Brice A, Dejean A, Sittler A: **SUMOylation attenuates the aggregation propensity and cellular toxicity of the polyglutamine expanded ataxin-7**. *Hum Mol Genet* 2010, **19**:181–195.

79. Poukka H, Karvonen U, Jänne OA, Palvimo JJ: **Covalent modification of the androgen receptor by small ubiquitin-like modifier 1 (SUMO-1)**. *Proc Natl Acad Sci* 2000, **97**:14145–14150.

80. Riley BE, Zoghbi HY, Orr HT: **SUMOylation of the polyglutamine repeat protein, Ataxin-1, is dependent on a functional nuclear localization signal**. *J Biol Chem* 2005, **280**:21942–21948.

81. Tsai YC, Fishman PS, Thakor NV, Oyler GA: **Parkin facilitates the elimination of expanded polyglutamine proteins and leads to preservation of proteasome function**. *J Biol Chem* 2003, **278**:22044–22055.

82. Cummings CJ, Reinstein E, Sun Y, Antalffy B, Jiang Y, Ciechanover A, Orr HT, Beaudet AL, Zoghbi HY: **Mutation of the E6-AP ubiquitin ligase reduces nuclear inclusion frequency while accelerating polyglutamine-induced pathology in SCA1 mice**. *Neuron* 1999, **24**:879–892.

83. Chu Y, Mickiewicz AL, Kordower JH: **α-synuclein aggregation reduces nigral myocyte enhancer Factor-2D in idiopathic and experimental Parkinson’s disease**. *Neurobiol Dis* 2011, **41**:71–82.

84. She H, Yang Q, Mao Z: **Neurotoxin-induced selective ubiquitination and regulation of MEF2A isoform in neuronal stress response**. *J Neurochem* 2012, **122**:1203–1210.

85. She H, Mao Z: **Regulation of myocyte enhancer factor-2 transcription factors by neurotoxins**. *NeuroToxicology* 2011, **32**:563–566.

86. González P, Álvarez V, Menéndez M, Lahoz CH, Martínez C, Corao AI, Calatayud MT, Peña J, García-Castro M, Coto E: **Myocyte enhancing factor-2A in Alzheimer’s disease: Genetic analysis and association with MEF2A-polymorphisms**. *Neurosci Lett* 2007, **411**:47–51.

87. Burton TR, Dibrov A, Kashour T, Amara FM: **Anti-apoptotic wild-type Alzheimer amyloid precursor protein signaling involves the p38 mitogen-activated protein kinase/MEF2 pathway**. *Brain Res Mol Brain Res* 2002, **108**:102–120.

88. Basso M, Giraudo S, Corpillo D, Bergamasco B, Lopiano L, Fasano M: **Proteome analysis of human substantia nigra in Parkinson’s disease**. *Proteomics* 2004, **4**:3943–3952.

89. Shim SM, Lee WJ, Kim Y, Chang JW, Song S, Jung Y-K: **Role of S5b/PSMD5 in proteasome inhibition caused by TNF-α/NFκB in higher eukaryotes**. *Cell Reports* 2012, **2**:603–615.

90. Ciechanover A, Brundin P: **The ubiquitin proteasome system in neurodegenerative diseases: Sometimes the chicken, sometimes the egg**. *Neuron* 2003, **40**:427–446.

91. Tyedmers J, Mogk A, Bukau B: **Cellular strategies for controlling protein aggregation**. *Nat Rev Mol Cell Biol* 2010, **11**:777–788.

92. Zuccato C, Cattaneo E: **Brain-derived neurotrophic factor in neurodegenerative diseases**. *Nat Rev Neurol* 2009, **5**:311–322.

93. Cao M, Tan X, Jin W, Zheng H, Xu W, Rui Y, Li L, Cao J, Wu X, Cui G, Ke K, Gao Y: **Upregulation of Ras homolog enriched in the brain (Rheb) in lipopolysaccharide-induced neuroinflammation**. *Neurochem Int* 2013, **62**:406–417.

94. Cheng H-C, Kim SR, Oo TF, Kareva T, Yarygina O, Rzhetskaya M, Wang C, During M, Talloczy Z, Tanaka K, Komatsu M, Kobayashi K, Okano H, Kholodilov N, Burke RE: **Akt suppresses retrograde degeneration of dopaminergic axons by inhibition of macroautophagy**. *J Neurosci Off J Soc Neurosci* 2011, **31**:2125–2135.

95. Zhang S, Binari R, Zhou R, Perrimon N: **A genomewide RNA interference screen for modifiers of aggregates formation by mutant huntingtin in *Drosophila***. *Genetics* 2010, **184**:1165 –1179.

96. Doumanis J, Wada K, Kino Y, Moore AW, Nukina N: **RNAi screening in *Drosophila* cells identifies new modifiers of mutant huntingtin aggregation**. *PLoS ONE* 2009, **4**:e7275.

97. Sang T-K, Li C, Liu W, Rodriguez A, Abrams JM, Zipursky SL, Jackson GR: **Inactivation of *Drosophila* Apaf-1 related killer suppresses formation of polyglutamine aggregates and blocks polyglutamine pathogenesis**. *Hum Mol Genet* 2005, **14**:357–372.

98. Bollimuntha S, Singh BB, Shavali S, Sharma SK, Ebadi M: **TRPC1-mediated inhibition of 1-methyl-4-phenylpyridinium ion neurotoxicity in human SH-SY5Y neuroblastoma cells**. *J Biol Chem* 2005, **280**:2132–2140.

99. Ryoo HD, Bergmann A, Gonen H, Ciechanover A, Steller H: **Regulation of *Drosophila* IAP1 degradation and apoptosis by reaper and ubcD1**. *Nat Cell Biol* 2002, **4**:432–438.

100. Baratchi S, Kanwar RK, Kanwar JR: **Survivin: A target from brain cancer to neurodegenerative disease**. *Crit Rev Biochem Mol Biol* 2010, **45**:535–554.

101. Voßfeldt H, Butzlaff M, Prüßing K, Ní Chárthaigh R-A, Karsten P, Lankes A, Hamm S, Simons M, Adryan B, Schulz JB, Voigt A: **Large-scale screen for modifiers of Ataxin-3-derived polyglutamine-induced toxicity in *Drosophila***. *PLoS ONE* 2012, **7**:e47452.
