# Supplementary figures and images for: NeuroGeM, a knowledgebase of genetic modifiers in neurodegenerative diseases
Source: BMC Med Genomics. 2013 Nov 14;6:52. doi: 10.1186/1755-8794-6-52 (PMC3833180; doi:10.1186/1755-8794-6-52)

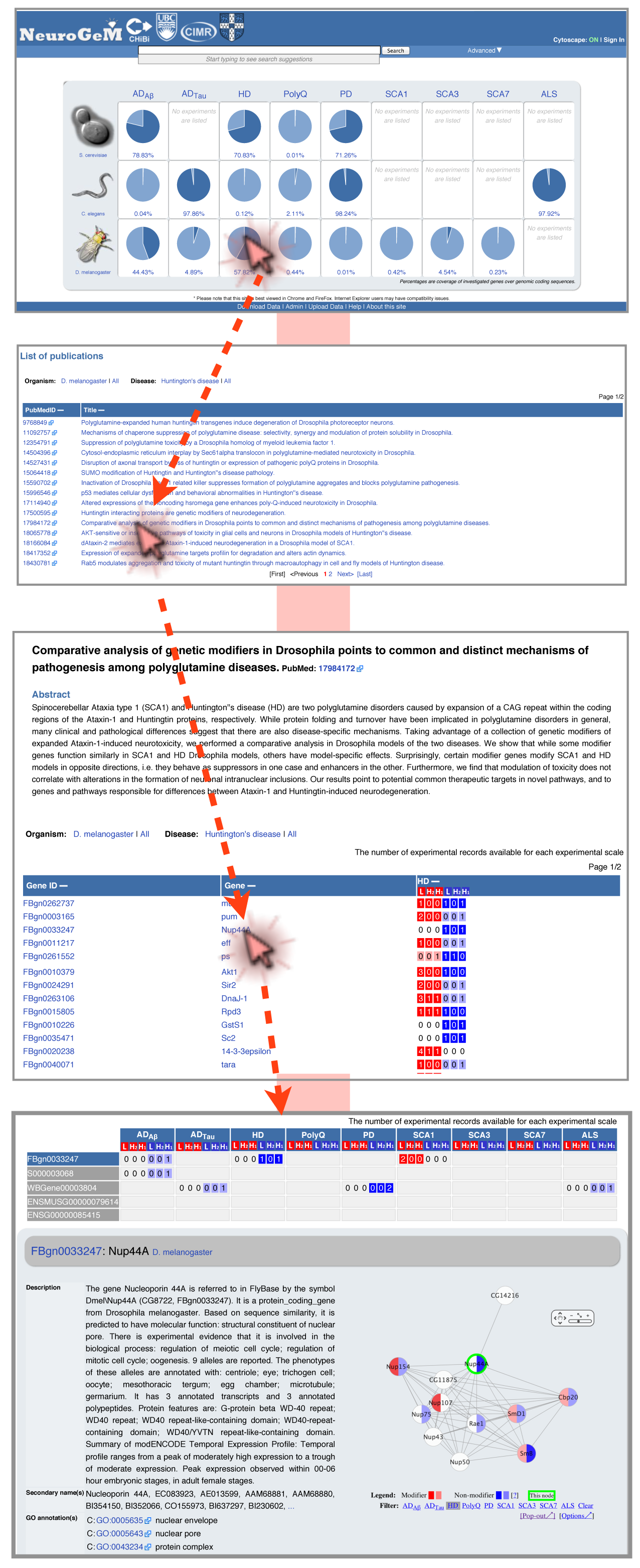

Supplement: Additional file 2: Figure S1 — A figure to illustrate a categorical search. [file 1755-8794-6-52-S2.tiff]

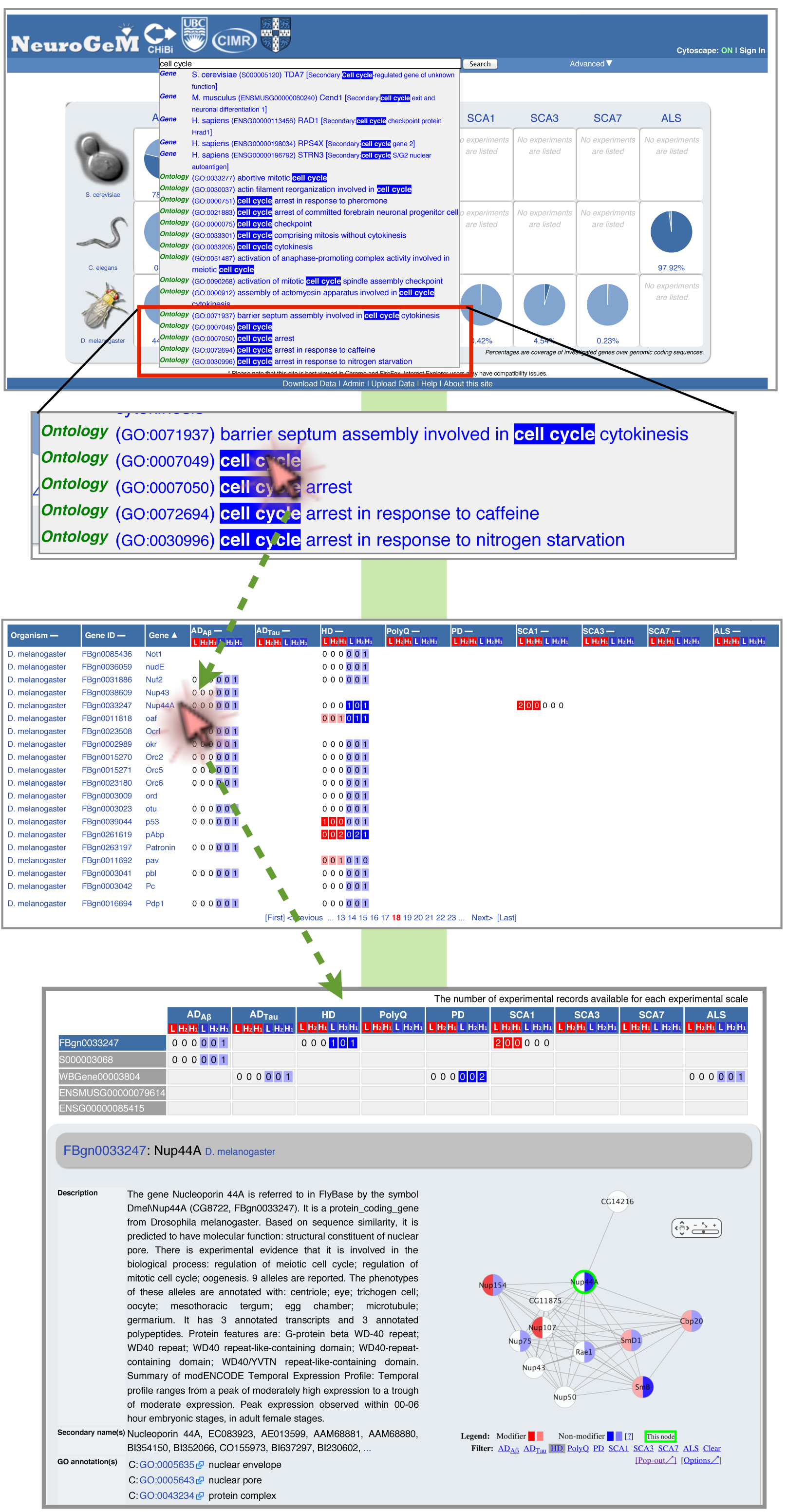

Supplement: Additional file 3: Figure S2 — A figure to illustrate an ontology-based search. [file 1755-8794-6-52-S3.tiff]
